# Best Practices and Considerations for Conducting Research on Diet–Gut Microbiome Interactions and Their Impact on Health in Adult Populations: An Umbrella Review

**DOI:** 10.1016/j.advnut.2025.100419

**Published:** 2025-04-01

**Authors:** Tatiana Diacova, Christopher J Cifelli, Cindy D Davis, Hannah D Holscher, Mary E Kable, Johanna W Lampe, Marie E Latulippe, Kelly S Swanson, J Philip Karl

**Affiliations:** 1 Graduate Group in Nutritional Biology, University of California Davis, Davis, CA, United States; 2 National Dairy Council, Rosemont, IL, United States; 3 Agricultural Research Service, United States Department of Agriculture, Beltsville, MD, United States; 4 Department of Food Science and Human Nutrition, University of Illinois Urbana-Champaign, Urbana, IL, United States; 5 Division of Nutritional Sciences, University of Illinois Urbana-Champaign, Urbana, IL, United States; 6 Division of Public Health Sciences, Fred Hutchinson Cancer Center, Seattle, WA, United States; 7 Institute for the Advancement of Food and Nutrition Sciences, Washington, DC, United States; 8 Department of Animal Sciences, University of Illinois Urbana-Champaign, Urbana, IL, United States; 9 Military Nutrition Division, U.S. Army Research Institute of Environmental Medicine, Natick, MA, United States

**Keywords:** systematic review, research methods, study design, gut microbiota, nutrition

## Abstract

Diet modulates gut microbiome composition and function. However, determining causal links between diet–gut microbiome interactions and human health is complicated by inconsistencies in the evidence, arising partially from variability in research methods and reporting. Widespread adoption of standardized best practices would advance the field but require those practices to be identified, consolidated, and discussed. This umbrella review aimed to identify recommended best practices, define existing gaps, and collate considerations for conducting research on diet–gut microbiome interactions and their impact on human health outcomes. Reviews meeting inclusion criteria and published after 2013 were identified using a systematic search. Recommendations, considerations, and gaps relating to the best practices associated with study design, participant selection, dietary intervention/assessment, biological sample collection, and data analysis and reporting were extracted and consolidated. Eight narrative reviews were included. Several general points of agreement were identified, and a recurring theme was that best practices are dependent upon the research aims, outcomes, and feasibility. Multiple gaps were also identified. Some, such as suboptimal diet assessment methods and lack of validated dietary intake biomarkers, are particularly relevant to nutrition science. Others, including defining a “healthy” gut microbiome and the absence of standardized sample and data collection/analysis protocols, were relevant specifically to gut microbiome research. Gaps specific to diet–gut microbiome research include the underrepresentation of microbiome-modulating dietary components in food databases, lack of knowledge regarding interventions eliciting changes in the gut microbiome to confer health benefits, lack of in situ measurement methods, and the need to further develop and refine statistical approaches for integrating diet and gut microbiome data. Future research and cross-disciplinary exchange will address these gaps and evolve the best practices. In the interim, the best practices and considerations discussed herein, and the publications from which that information was extracted provide a roadmap for conducting diet–gut microbiome research.

This trial was registered at PROSPERO as CRD42023437645.

**Statement of Significance**

This umbrella review aims to help standardize methods and reporting in research on diet–gut microbiome interactions and their impact on human health outcomes by identifying current recommended best practices, defining existing gaps where the best practices are lacking, and collating considerations for conducting research in the field. In so doing, the review provides guidance for conducting diet–gut microbiome research and identifies several specific priority areas for addressing gaps relevant to establishing the best practices.

## Introduction

The human gut microbiome encompasses a dynamic community of organisms including prokaryotes, eukaryotes, archaea, and fungi residing in the gastrointestinal tract. Bacteria are the most abundant and most studied domain with *Bacteroidota* (previously *Bacteroidetes*), *Bacillota* (previously *Firmicutes*), *Actinomycetota* (previously *Actinobacteria*), *Pseudomonadota* (previously *Proteobacteria*), *Verrucomicrobiota* (previously *Verrucomicrobia*), and *Fusobacteriota* (previously *Fusobacteria*) phyla predominating [1]. Evidence suggests that gut microbes contribute to various host physiological functions throughout the body including maturation and modulation of the immune system [2], maintenance of gut barrier health [3], and regulation of brain function and behavior [4] among others. Accordingly, the gut microbiome is increasingly recognized as a critical mediator of human health and disease risk [[5], [6], [7], [8], [9], [10]].

Diet is one important factor affecting gut microbiome composition, function, and metabolic activity [1,[11], [12], [13], [14], [15], [16]] and may explain as much as 20% of interindividual variation in human gut microbiome composition [17]. Dietary components impact the gut microbiome in several ways. These include providing substrate for microbial metabolism, directly or indirectly altering the gastrointestinal environment, and influencing microbial interactions within the community [18]. Gut microbes, in turn, modulate the digestion, bioavailability, and absorption of various dietary components [[19], [20], [21], [22]]. This bidirectional relationship results in the production of metabolites and other compounds that play an important role in host health and disease by altering the function of biological systems throughout the body from the gut to the brain [23].

Despite evidence supporting a strong connection between diet, the gut microbiome and human health, conflicting results regarding specific diet–gut microbiome interactions and their influence on host health are widespread [24]. Reasons for the inconsistencies vary but may be caused in part by the diverse approaches used for studying diet–microbiome interactions and their impact on health outcomes. The use of diverse research methods is attributable in part to a rapidly evolving field in which new technologies and techniques are constantly being developed. However, these advancements, while allowing for new insights into diet–gut microbiome interactions, complicate comparisons across studies and make drawing definitive conclusions difficult. The resulting uncertainty impedes the development of evidence-based dietary recommendations aimed at nourishing the gut microbiome to improve human health.

Widespread adoption of standardized best practices for studying the role of diet–microbiome interactions in human health would facilitate the comparison and integration of studies ultimately needed to develop evidence-based recommendations. However, doing so first requires the best practices and considerations for study design and conduct to be identified, consolidated, and discussed. Narrative reviews focused on the best practices for the design of gut microbiome [[25], [26], [27], [28], [29], [30], [31], [32], [33], [34], [35], [36], [37], [38], [39], [40], [41]] and nutrition studies exist [[42], [43], [44], [45]]. Others have specifically considered the best practices for study design in research focused on diet–gut microbiome interactions and human health [[46], [47], [48], [49], [50], [51], [52], [53]]. However, among those reviews, foci and recommendations have differed. This umbrella review therefore aimed to identify current recommended best practices in diet–gut microbiome research, define existing gaps where the best practices have not been identified, and collate considerations for the design of studies aiming to determine effects of diet–gut microbiome interactions on human health-related outcomes. The overarching goal of this work is to improve study design and reporting to facilitate synthesis and comparison of results across studies to ultimately advance understanding of the causal effects of diet–microbiome interactions on human health and disease.

## Methods

The protocol for this umbrella review was developed according to the Joanna Briggs Institute Manual for Evidence Synthesis for Umbrella Reviews and was registered on Open Science Network (https://doi.org/10.17605/OSF.IO/FUPZS) and PROSPERO (CRD42023437645). The central question of the review was: what are the established and/or suggested best practices and considerations for study design and research methods in human studies exploring the effects of nutrition interventions on gut microbiome-related health outcomes? The specific objectives were to identify perspectives, reviews, systematic reviews, and meta-analyses exploring the best practices and considerations in diet–microbiome research; consolidate, summarize, and discuss the best practices and study design considerations; and identify gaps where the best practices have not been established.

### Search strategy

Two separate literature searches were conducted using PubMed on 24 February, 2023, and 3 March, 2023, respectively, and then duplicated on 6 October, 2023. The search was restricted to manuscripts published in the past 10 y. Manual search of reference lists from identified articles and personal knowledge of the authors was used to identify additional relevant publications.

#### Search 1

(microbiome[Title] or microbiota[Title] or microflora[Title]) AND (gut[Title] OR intestinal[Title] OR gastrointestinal[Title] OR enteric[Title] OR diet∗[Title] OR nutrition∗[Title] OR prebiotic∗[Title] OR probiotic∗[Title] OR fiber∗[Title]) AND (“best practices”[Title] OR challenge∗[Title] OR “study design”[Title] OR recommendation∗[Title] OR guide∗[Title] OR consideration∗[Title] OR guideline[Title])

#### Search 2

(microbiome[Title] or microbiota[Title] or microflora[Title]) AND (gut[Title] OR intestinal[Title] OR gastrointestinal[Title] OR enteric[Title] OR diet[Title] OR nutrition[Title] OR “diet therapy”[mesh]) AND (“best practices”[Title] OR challenge∗[Title] OR “study design”[Title] OR recommendation∗[Title] OR guide∗[Title] OR consideration∗[Title])

### Eligibility criteria

Only narrative reviews, systematic reviews, meta-analyses, consensus statements, perspectives, and commentaries published in the last 10 y (2013–2023) were included. Articles were required to include recommendations for methods related to randomized controlled trials (RCTs) or nonrandomized study designs relevant to any nonpatient human population and discuss methods or study design considerations relating to both nutrition-related interventions and gut microbiome-related outcomes. Gut microbiome-related outcomes included community composition, microbiome-derived compounds, food-derived compounds altered by the gut microbiome, gut microbiome function, and gut microbiome-mediated health effects.

Exclusion criteria included the absence of discussion on the best practices, standardization, considerations, or gaps relating to the best practices and method standardization; focus on design of animal, in vitro or in silico studies or design of studies for strictly human infant or patient populations; focus on microbiome populations in locations other than the gastrointestinal tract; and focus on interventions that are not nutrition related. Publications discussing the development of a new method or dietary therapy and publications not including methodological considerations relating to both nutrition and the gut microbiome were excluded.

### Screening and data extraction

Abstracts were screened for relevancy using Abstrakr software [54] by 2 independent reviewers (TD and JPK) blinded to each other’s responses. The full text was retrieved for all abstracts considered as maybe or definitely relevant by ≥1 reviewer. Full texts were then screened by the same 2 independent reviewers who were blinded to each other’s responses and according to the eligibility criteria described previously. Discrepancies were resolved through discussion.

Data were extracted from all manuscripts meeting the inclusion/exclusion criteria by 2 independent reviewers (TD and JPK) using the same data extraction template. The template was in the form of a spreadsheet organized by the predetermined structural review categories and subcategories of interest. The first category focused on study design and participant selection. Subcategories included research questions and aims, trial design, blinding, duration (run-in, intervention, washout), effect size, sample size calculations, statistical analysis plan, addressing interindividual variability, inclusion/exclusion criteria and important metadata to collect. The second category focused on diet intervention and assessment. Subcategories included intervention design and development (diets and foods, supplements, dose, and placebo/control), assessing adherence/compliance (biomarkers, ensuring compliance), describing diet/intervention, diet standardization, methods (emerging methods, commonly used methods), database selection (from nutrition- to gut microbiome centered), and other considerations (timing with microbiome analysis, timing and location of meal/supplement consumption). The third category described biological sample analysis. Subcategories within this category were sample collection (types of sample(s) quantity, frequency, timing, collection/transport/storage/processing), fecal sample data to record, and microbiota analysis (measurement methods, relative compared with absolute abundance, diversity measures, and reporting taxonomy). The last category pertained to diet–microbiome data analysis, integration and reporting and included the subcategories microbiome data transformation and normalization, statistical modeling (general characteristics, diet–microbiome data integration), correction for multiple comparisons, interpreting and reporting of results, and data sharing.

Text related to the categories/subcategories was extracted and coded as a “best practice,” “consideration,” or “gap in knowledge.” Extracted text was then compared between the 2 reviewers and consolidated if in agreement. Discrepancies were resolved through discussion.

### Review quality

Methodological quality for narrative reviews, perspectives, and opinions was assessed by 2 blinded reviewers (TD and JPK) using the Joanna Briggs Institute Critical Appraisal Checklist for Text and Opinion Papers (Supplemental Table 1). Disagreements were resolved through discussion.

### Role of the expert group

Conceptualization and design of the umbrella review were informed by all authors who comprised a panel of scientists with expertise in diet–gut microbiome interactions formed by the Institute for the Advancement of Food and Nutrition Sciences. The group provided feedback on the review protocol before registration on Open Science Network/PROSPERO and reviewed extracted results relating to the best practices, considerations and gaps in knowledge. In several cases, expert group opinion differed from the extracted information. Those differences are noted in the text and tables.

### Summary and presentation of extracted information

Review results were organized and presented according to the structural review categories defined previously. A summary of the extracted best practices for each category is described in the main text of the results. Summaries of extracted considerations and knowledge gaps are provided in the Supplemental Results. For each category, we also include a “summary of points of agreement and knowledge gaps” table and a “synopsis of best practice recommendations” figure to further summarize and consolidate extracted information. “Points of agreement” for the purpose of this manuscript were defined as the best practices for which a majority of articles commenting on a particular category were in agreement on the best practice without another review stating something contradictory. Both explicit identification of the best practice and inclusion of statements that allowed the best practice to be inferred from the authors’ discussion were used to determine agreement.

## Results

**FIGURE 1:**
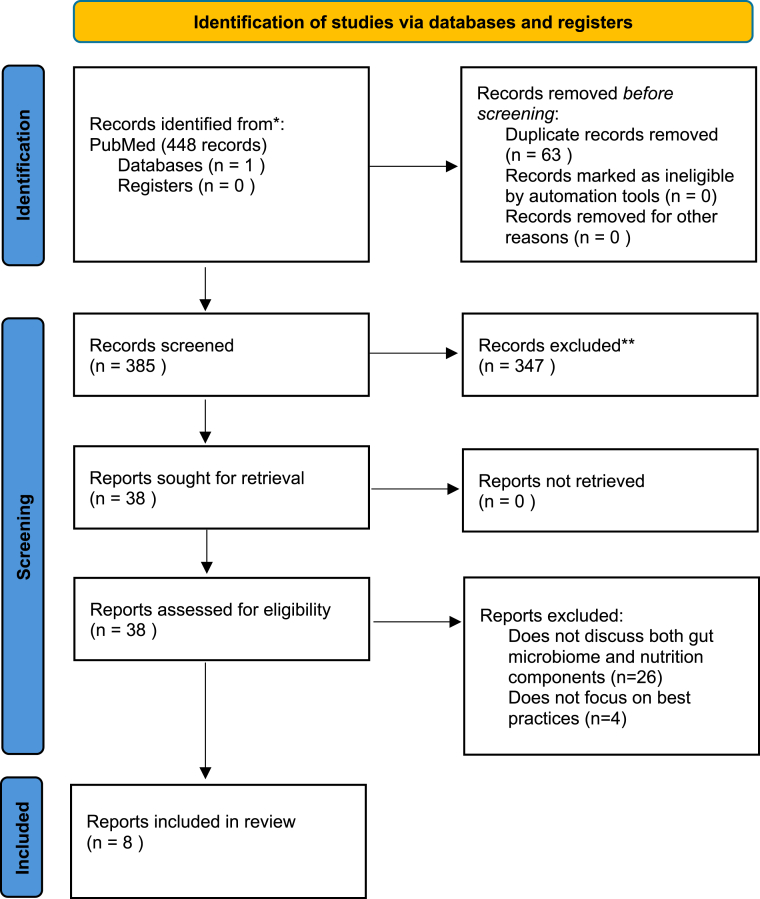
PRISMA diagram.

The PubMed search identified 365 unique reports for screening (Figure 1). Of those, 38 abstracts were selected for full-text review, and 8 publications were included in this review [[46], [47], [48], [49], [50], [51], [52], [53]]. Countries represented in authorship lists included the United States, Australia, the United Kingdom, New Zealand, Ireland, Finland, Serbia, Switzerland, France, Belgium, and Italy.

Although all 8 publications generally focused on the best practices, considerations, and gaps in knowledge for diet–gut microbiome studies, each presented a unique aspect. Choi et al. [46] discussed approaches to applying computational models for diet–gut microbiome data integration. Hughes et al. [47] focused on the development of precision nutrition in the context of diet–gut microbiome interactions. Klurfeld et al. [49] mainly focused on the best practices and considerations for studies with fiber as the dietary intervention, whereas Mohr et al. [51] discussed the best practices for RCTs of probiotics in physically active populations. Marques et al. [50] presented guidelines for conducting gut microbiome studies in experimental and clinical hypertension specifically. Swann et al. [53], aside from discussing the best practice guidelines for human gut microbiome intervention studies, also presented the European Union legislative aspects in relation to foods and health claims. Finally, Johnson et al. [48] and Shanahan et al. [52] provided a general overview of current practices in diet–gut microbiome research, while also suggesting several best practices.

### Study design and participant selection

**FIGURE 2:**
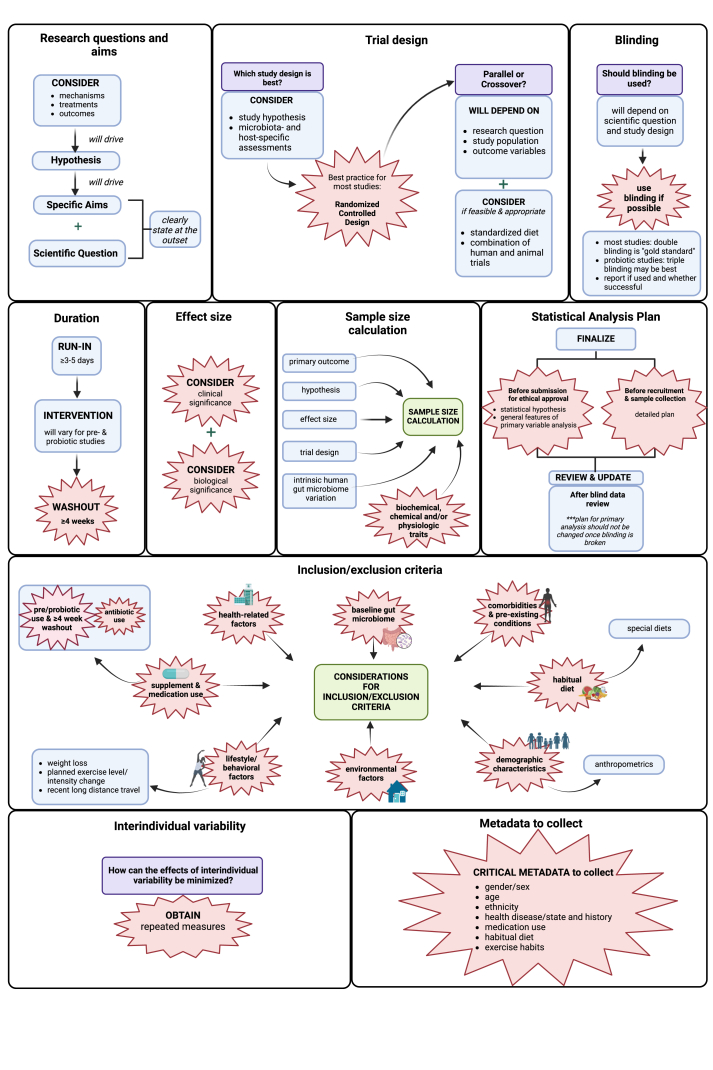
Synopsis of the best practice recommendations for study design and participant selection reported in diet–gut microbiome literature. Explosion boxes show points of agreement. Blue boxes show the best practice suggestions that were not points of agreement. Purple boxes show questions related to specific elements/subelements and green boxes show the names of subelements. Arrows depict a subelement leading or contributing to another subelement. Brackets show multiple best practice recommendations that apply to the same subelement. Created in BioRender. Diacova, T. (2025) https://BioRender.com/v70a688; https://BioRender.com/o69h346.

A summary of the results for the study design and participant selection category is provided in Table 1 [[47], [48], [49], [50], [51], [52], [53]], Figure 2, and Supplemental Table 2. A summary of considerations and knowledge gaps is available in the Supplemental Results.

**TABLE 1:** Summary of points of agreement and knowledge gaps in diet–gut microbiome literature: study design and participant selection.

Category	References	Points of agreement	Gaps
Research question and aims	[[51], [52], [53]]	None	None
Trial design	[[47], [48], [49], [50], [51], [52], [53]]	Randomized controlled trials	Best practices for integrating animal studies with human trials to establish causal relations and mechanisms.
Blinding	[48,50,51,53]	Blinding should be used.	None
Duration			
*Run-in*	[48]	None	Need for a run-in period and, if required, the optimal duration.
*Intervention*	[50,51]	None	• Duration of intervention required to demonstrate microbiome-mediated changes in host phenotype is often unknown. • Duration of intervention needed to elicit long-term or permanent changes in gut microbiome is unknown.
*Washout*	[[48], [49], [50], [51]]	≥4 wk to prevent carryover effects.	Duration of washout period required for any intervention effects on the gut microbiome to return to baseline may vary by intervention type and duration.
Effect size	[47,50]	Clinical and biological significance should both be considered.	• Biologically meaningful effect sizes for changes in gut microbiome features often unknown. • Often not possible to select beforehand which bacteria are expected to be modulated by a dietary intervention. • No current consensus on definition of a normal or abnormal microbiome making it difficult to determine if an increase or decrease of specific microbiome groups is any indication of any specific health effect.
Sample size calculations	[47,50,52,53]	Consider biochemical, chemical, and/or physiological traits.	Gut microbiome features (if any) on which sample size calculations should be based are often unknown.
Statistical analysis plan	[52,53]	Should be written before data collection.	None
Interindividual variability	[48,50,52]	Repeated measurements help reduce impact of interindividual variability.	None
Inclusion and exclusion criteria (factors to consider)			
*General considerations*	[48,50,51,53]	Inclusion and exclusion criteria should consider a variety of factors such as baseline microbiome composition, habitual diet, demographics characteristics, health-related factors, supplement and medication use, habitual diet, lifestyle/behavioral and environmental factors.	Specific guidance on how baseline microbiome composition and habitual diet should be addressed is lacking.
*Demographics/anthropometrics*	[50]	None	None
*Consumption of -biotics*	[50,51,53]	Consumption of pro/prebiotics in habitual diet should be considered and a ≥4wk washout period implemented.	Whether a washout from intake of other -biotics (e.g. postbiotics, synbiotics) is needed is not always clear.
*Washout for -biotics*	[50,51]		
*Medication use*	[50,51]	Antibiotic use should be considered.	• Effects of many medications on the microbiome are largely unknown. • Requirements for washout or need to account for other specific medications besides antibiotics are not defined.
*Washout for medication use*	[50,51]	None	
*Other*	[50,51,53]	Comorbidities and pre-existing conditions should be considered.	
Metadata to collect	[[48], [49], [50], [51], [52], [53]]	Critical metadata to collect are sex/gender, age, ethnicity, health/disease state and history, medication use, habitual diet, exercise habits.	Critical metadata to collect and include as covariates in analyses (or account for in participant selection and/or randomization) are undefined.
Inclusion of diverse population	[51]	None	Importance of inclusion of diverse population not being discussed as the best practice.

#### Research questions and aims

Three publications [[51], [52], [53]] discussed the best practices related to developing research questions and aims, but no points of agreement were identified. One publication [51] stated that mechanisms, treatments, and outcomes of interest are all important to consider when developing research questions and aims. The other 2 publications separately emphasized that the question and aims should be hypothesis driven [52] and clearly stated at the outset [53].

#### Trial design and blinding

Seven publications [[47], [48], [49], [50], [51], [52], [53]] discussed the best practices for trial design. A point of agreement was that RCT designs are optimal for most diet–gut microbiome studies (4 studies [47,49,51,53]). Two publications recommended crossover over parallel group designs [48,50], whereas another described parallel group designs as often being more suitable for certain interventions like probiotics [51]. Four publications discussed the best practices for blinding [48,[50], [51],^53^]. A point of agreement was that blinding should be used (3 studies [48,51,53]). Other recommendations made in single publications were that study designs be hypothesis driven [52], include both microbiome- and host-specific assessments [53], and may need to include both humans and animal models [49].

#### Duration of run-in, washout, and intervention

Best practices for the duration of run-in, washout, and intervention periods in diet–gut microbiome research were discussed by 1, 2, and 4 publications, respectively [[48], [49], [50], [51]]. The 1 publication that mentioned run-in duration [48] stated that the run-in period should be ≥3–5 d based on the evidence that gut microbiome can change as soon as 1 d after the dietary intervention has reached the distal gut [55]. In the 2 publications that discussed intervention durations [50,51], 1 noted that intervention durations should be 2–3 wk to allow the microbiome to stabilize [50], whereas another stated that intervention duration will depend on primary study outcomes and may require 4–16 wk for probiotic studies [51]. A point of agreement was identified regarding washout duration for crossover studies. Three of 4 publications indicated 4 wk as an appropriate washout duration (3 studies; [[49], [50], [51]]) though the minimum duration recommended ranged from 2 [49,51] to 4 [50] wk.

#### Effect size and sample size calculations

Five publications [47,48,50,52,53] discussed the best practices for effect size and sample size calculations. Points of agreement were that sample size and effect size determination should consider biochemical, chemical, and/or physiological traits (3 of 4 studies; [47,50,53]) as well as their biological and clinical significance (2 of 2 studies; [47,50]), respectively. Additional recommendations specific to sample size included conducting hypothesis-driven calculations ideally based on data from previous trials (1 publication; [53]) and accounting for intrinsic human gut microbiome variation (1 publication; [50]). Though 1 publication suggested that as many as 400–500 participants are needed for case-control and cross-sectional studies [48], no specific recommendations were given for experimental studies.

#### Statistical analysis plan

Two publications suggested the best practices for statistical analysis plans [52,53]. A point of agreement was that statistical analysis plans should include a general description of how the primary outcomes will be analyzed and should be developed before submission to review boards for approval [52,53]. One publication stated that the statistical analysis plan can be revised as a part of blind data review when indicated but that the plan should not be changed after blinding is broken [53].

#### Interindividual variability

Three publications [48,50,52] discussed the best practices for accounting for the marked interindividual variability observed across human gut microbiomes. A point of agreement recommends increasing sample size and collecting repeated samples [48,52] to reduce the impact of interindividual variability. One publication instead recommended using baseline microbiome composition as a control in analyses [50].

#### Inclusion and exclusion criteria (factors to consider)

Four publications discussed the best practices for developing study inclusion and exclusion criteria [48,50,51,53]. General points of agreement among all 4 reports were that these criteria should consider a variety of factors such as baseline microbiome composition, habitual diet, demographic characteristics, health-related factors, supplement and medication use, habitual diet, lifestyle/behavioral and environmental factors. Specific points of agreement were that baseline microbiome composition and habitual diet should be considered (3 publications [48,51,53]). Additional points of agreement were that pro-/prebiotic consumption in the habitual diet, antibiotic use, pre-existing health conditions, and comorbidities should all be considered [50,51,53]. Three publications discussed -biotic use, specifically mentioning probiotics (3 publications; [50,51,53]), prebiotics (2 publications; [51,53]), and fermented foods (1 publication; [51]). A point of agreement identified 4 wk as an appropriate washout period from those products (2 of 3 publications [50,51]). In contrast, there was no agreement in terms of the appropriate washout period from medication use, with recommendations ranging from 3–4 wk (1 publication; [51]) to 3–6 mo (1 publication; [50]). Defining age range of interest [53], considering demographics and anthropometric characteristics [50], maintaining temporal, geographical, and demographic consistency in participant recruitment for case-control study designs [50] and including a diverse participant population [51] were also recommended as the best practices in separate individual publications. Other factors mentioned by a single publication as important to consider for exclusion criteria were recent weight loss, special diets, plans to change exercise habits, and recent long-distance travel [53].

#### Metadata to collect

Six publications [[48], [49], [50], [51], [52], [53]] discussed the best practices for metadata to collect within the general categories of: demographics and anthropometrics, overall and gastrointestinal health-related factors, supplement and medication use, diet, lifestyle, geography, and environment. Overall, points of agreement were that the critical metadata to collect include sex/gender (4 publications; [[48], [50], [51], [53]]), age (4 publications; [[48], [50], [51], [53]]), ethnicity (4 publications; [[48], [50], [51], [52]]), health/disease history (4 publications; [[48], [51], [52], [53]]), medication use (4 publications; [[48], [50], [52], [53]]), habitual diet (4 publications; [[48], [50], [51], [53]]), and exercise habits (4 publications; [[48], [49], [51], [53]]). Additional recommendations extracted from 1 or more publications for metadata to collect can be found in Supplemental Table 2.

### Diet intervention and assessment

**FIGURE 3:**
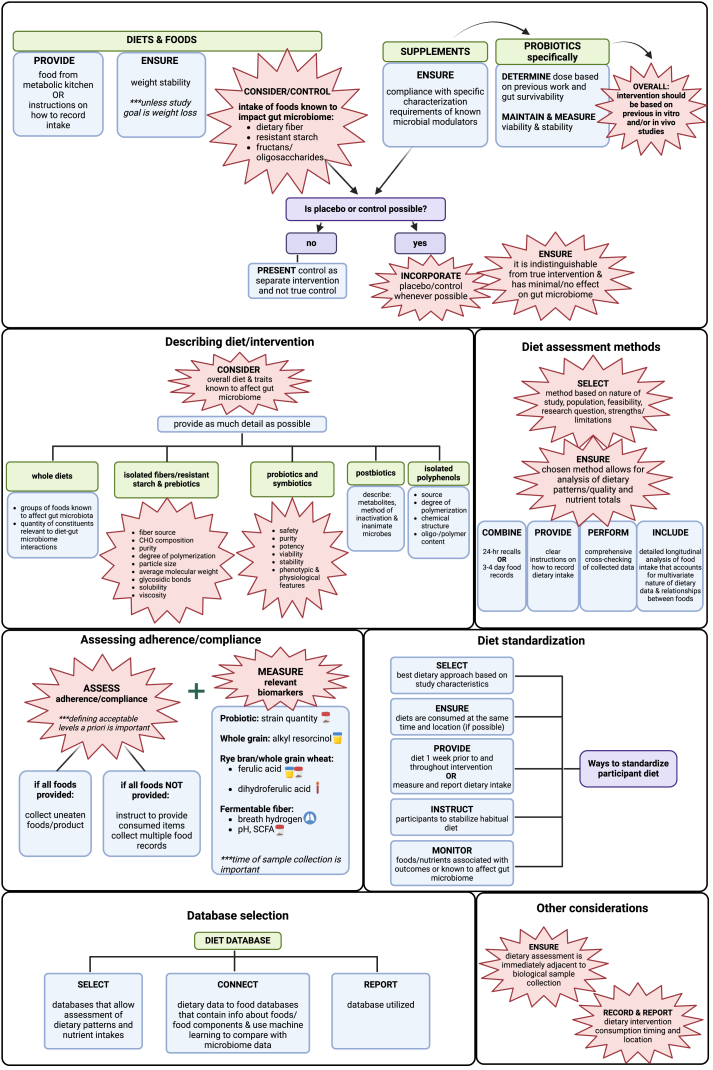
Synopsis of the best practice recommendations for diet intervention and assessment reported in diet–gut microbiome literature. Explosion boxes show points of agreement. Blue boxes show the best practice suggestions that were not points of agreement. Purple boxes show questions related to specific elements/subelements and green boxes show names of subelements. Arrows depict subelement leading or contributing to another subelement. Brackets show multiple best practice recommendations that apply to the same subelement. Created in BioRender. Diacova, T. (2025) https://BioRender.com/j99x044; https://BioRender.com/m62d845.

A summary of the results for the diet intervention and assessment category is provided in Table 2 [[46], [47], [48], [49], [50], [51], [52], [53]], Figure 3, and Supplemental Table 3. A summary of extracted considerations and knowledge gaps is available in the Supplemental Results.

**TABLE 2:** Summary of points of agreement and knowledge gaps in diet–gut microbiome literature: diet intervention and assessment.

Category	References	Points of agreement	Gaps
Intervention design and development			
*Diets and foods*	[49,50,53]	Control intake of foods known to impact microbiome (e.g. fiber, resistant starch, fructans, and other oligosaccharides).	• To what extent factors like food structure, preparation method, delivery mode and matrix should be considered need to be determined. • Best strategies for designing intervention and placebo/control to isolate diet–microbiome interaction as causal factor driving changes in host phenotype (vs. microbiome-independent effect of the intervention) are needed. • 3D structures of dietary fibers are not uniform, have not been completely described and may impact diet–gut microbiome interactions. • Often unclear if dose should be based on absolute dose, energy intake or other factors. • Minimum dose required to elicit effects on gut microbiome may not be known. • Ideal placebos for all interventions are not defined.
*Supplements*	[51,53]	Intervention should be based on previous in vitro and/or in vivo studies.	
*Dose*	[51]	None	
*Placebo/control*	[47,48,50,51,53]	Include placebo that, when possible, is indistinguishable from intervention and has minimal/no impact on the gut microbiome.	
Describing diet/intervention	[46,48,49,52,53]	Overall diet should be considered. When describing intervention attention needs to be given to characteristics that would influence any interaction with the gut microbiome. These characteristics will vary based on the intervention.	Given the myriad dietary components that may impact the gut microbiome, the level of detail required to ensure reproducibility is unclear and minimal/optimal reporting guidelines are not available.
Diet standardization	[48,50,51,53]	None	• Whether standardizing or stabilizing diet offers the best approach to reducing/controlling impact of interindividual variability. • Level of diet standardization/control required to definitively elucidate the effects of diet–microbiome interactions on health outcomes.
Assessing adherence/compliance			
*Biomarkers*	[[49], [50], [51], [52], [53]]	Measure biomarkers when feasible.	Few, if any, established biomarkers for diet–microbiome and microbiome–host interactions exist. Future research needed to refine existing and identify new biomarkers (52).
*Ensuring compliance*	[48,50,51,53]	Compliance should be measured with method used varying based on study design.	Acceptable level of compliance to demonstrate efficacy is undefined.
Diet assessment methods			
*Emerging methods*	[48]	None	• Commonly used existing methods may fail to account for/collect enough detail on dietary factors that may impact the gut microbiome or may be sufficiently inaccurate to limit their applicability. • Tools that facilitate less onerous, yet accurate estimation of dietary intake is needed. • There is a need for the further development of methods that enable measuring nutrients in foods that are relevant to microbes and methods that estimate the availability of nutrients at the luminal substrate–microbe interface. • Connecting food servings to the quantity of food that reaches the distal colon where it can ultimately impact the gut microbiome will require new approaches to dietary assessment and analysis.
*Commonly used methods*	[46,48,[51], [52], [53]]	Multiple options available and choice depends on study design/aims. Should follow the best practice recommendations reported elsewhere. Method should allow for analysis of dietary patterns/quality in addition to single nutrients if relevant for outcomes of interest.	
Database selection	[48,53]	None^1^	• No single existing database appears to capture all information on dietary factors that may impact diet–microbiome interactions. • A shared food ontology that can harmonize dietary data collected in different global regions and by different tools is needed but does not yet exist [48].
Other considerations			
*Timing with microbiome analysis*	[48,52]	Dietary data collection should be timed to coincide with microbiome measurement.	
*Timing and location of meal/supplement consumption*	[48,50,51]	Record and report timing and location of consumption.	Unclear how to account for variability in timing/location of meal consumption in data analysis.

1 Expert group opinion: although there is no agreement on the best database to use, it is essential to report which database was used.

#### Intervention design and development

Six publications [[47], [48], [49], [50], [51],^53^] provided the best practice recommendations for designing intervention diets and foods, supplements and doses, and developing appropriate placebos/controls. Three of those publications provided the best practice recommendations for designing diets and foods. A point of agreement among all 3 was that dietary components known to impact the gut microbiota (e.g. fiber, resistant starch, fructans, and other oligosaccharides) should be considered when designing intervention and control diets [49,50,53]. A single publication recommended that diets should be designed to ensure weight stability when weight loss is not an outcome [50].

Three of the publications also discussed the development of placebos and controls. A point of agreement was that the placebo or control diet should be indistinguishable from the intervention when possible and have minimal or no impact on the gut microbiome [47,51,53]. For studies of isolated dietary fiber interventions, microcrystalline cellulose was recommended as an ideal placebo (1 publication; [53]).

Finally, 2 publications [51,53] specifically discussed the best practice recommendations for -biotic studies. Both agreed that the intervention should be based on in vitro, preclinical and/or human studies and be thoroughly characterized. One publication [51] stated that probiotic studies should focus on describing the strains, species and strain designations. The other publication [53] emphasized the importance of ensuring compliance with specific characterization requirements of known microbial modulators when using probiotics, prebiotic fibers, and polyphenols as intervention. Both papers also commented on the importance of viability and stability of any probiotic intervention, stating that the product should be maintained in a live state throughout the study period (1 publication; [51]) and viability and stability should be monitored and documented (1 publication; [53]).

#### Describing diets and interventions

Six publications [46,48,49,[51], [52], [53]] discussed the best practices for describing diets and interventions. Four of those publications discussed the best practices related to whole foods and diets. A point of agreement was that studies should provide information on the overall diet and detailed information regarding specific individual nutrients or foods being tested should be reported (3 publications; [46,49,52]). Two of the publications specifically recommended that foods known or suspected to impact the microbiota should be reported when possible [48,52]. Other recommendations included providing as much detail as possible when describing any type of dietary intervention (1 publication; [49]).

Related to dietary compounds with the potential to interact with gut microbes, 2 publications provided recommendations regarding details to describe in interventions using isolated fibers, resistant starches, and prebiotics [49,53]. Points of agreement were that those details should include fiber source, carbohydrate composition, purity, degree of polymerization, particle size, average molecular weight and distribution range, glycosidic bonds, solubility, and viscosity of the compound. Confirming resistance to digestion was a point of agreement as well (2 publications; [49,53]). Similarly, the single publication providing recommendations for describing interventions using isolated polyphenols identified polyphenol source, degree of polymerization, chemical structure, and oligo-/polymer content as important characteristics to describe [53].

Two publications provided recommendations regarding details to describe interventions using probiotic and synbiotic interventions. Points of agreement were that phenotypic and physiological characteristics of the organisms used should be described and the safety, purity, potency, viability, and stability of the interventions should be reported (2 publications; [51,53]). Regarding other -biotic interventions, 1 publication recommended reporting the method of inactivation and characterization of metabolites and inanimate microbes for studies testing postbiotic interventions [51].

#### Diet standardization

Four publications recommended the best practices for diet standardization [48,50,51,53] but no points of agreement were identified. Recommendations included providing diets when possible (1 publication; [51]), attempting to stabilize participants’ diets (2 publications; [48,53]) and choosing the best approach based on study aims and outcomes (1 publication; [48]). One publication also recommended standardizing the time and location of consumption of provided diets [48].

#### Assessing adherence/compliance

Six publications discussed the best practices for assessing intervention adherence/compliance [[48], [49], [50], [51], [52], [53]]. Points of agreement were that compliance measures should be incorporated into the study (6 publications; [[48], [49], [50], [51], [52], [53]]) and that biomarkers should be used to assess compliance metrics when feasible (5 publications; [48,[50], [51], [52], [53]]). Recommended methods of assessing compliance included collecting multiple food records and any uneaten provided foods and intervention products (2 publications; [48,50]). Two publications recommended that acceptable levels of compliance should be determined a priori, and noncompliant participants excluded from analyses [48,53].

#### Diet assessment methods

Five publications discussed the best practices for diet assessment methods [46,48,[51], [52], [53]]. Points of agreement were that multiple options are currently available, that the selection of the optimal method to use will depend on the study design and the selected method should be based on the nature of study, participant population, feasibility, and strengths/limitations of the method in relation to the research question (3 publications; [[51], [52], [53]]). An additional point of agreement among the reports was that the chosen method should allow for analysis of dietary patterns or quality (3 publications; [46,48,52]). One publication recommended using technology and biochemical markers for diet assessment, if possible [48] whereas 2 publications recommended combining multiple 24-h recalls [e.g. USDA Automated Multiple-Pass Method via Automated Self-Administered Dietary Assessment Tool 24] or 3–4-d food records and food frequency questionnaires [48,51]. Additional recommendations included providing study participants with clear instructions and training on how to include the level of detail necessary before completion of diet recording (2 publications; [48,52]) and performing comprehensive cross-checking of collected data (1 publication; [52]).

#### Database selection

Two publications discussed dietary database selection [48,53]. No points of agreement were identified. One publication recommended that the diet database utilized be reported [53]. Another publication discussed several characteristics of a database that would be suitable for a diet–gut microbiome study, such as extensive information about foods and food components to enable the use of machine learning approaches to link dietary information and gut microbiome variables [48].

#### Other considerations

Four publications discussed the best practices for other categories relevant to dietary data collection and analysis [48,[50], [51], [52]]. Within the topics discussed, points of agreement included timing dietary data collection to coincide with microbiome measurements (2 publications; [48,52]) and recording and reporting the timing and location of dietary intervention consumption (3 publications; [48,50,51]). Finally, 1 publication recommended collecting 2–3 d of food records before each microbiome measurement [48].

### Biological sample analysis

**FIGURE 4:**
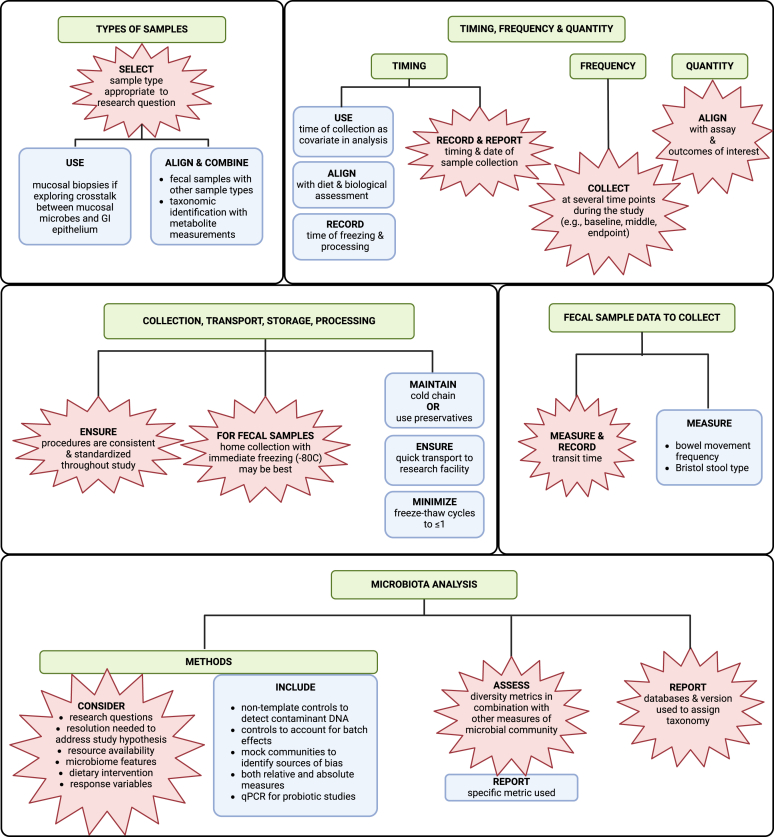
Synopsis of the best practice recommendations for biological sample analysis reported in diet–gut microbiome literature. Explosion boxes show points of agreement. Blue boxes show the best practice suggestions that were not points of agreement. Green boxes show the names of subelements. Arrows depict subelement leading or contributing to another subelement. Brackets show multiple best practice recommendations that apply to the same subelement. Created in BioRender. Diacova, T. (2025) https://BioRender.com/z80h470.

A summary of the results for biological sample analysis category is provided in Table 3 [47,48,[50], [51], [52], [53]], Figure 4, and Supplemental Table 4. A summary of considerations and knowledge gaps is available in the Supplemental Results.

**TABLE 3:** Summary of points of agreement and knowledge gaps in diet–gut microbiome literature: biological sample analysis.

Category	References	Points of agreement	Gaps
Bio sample collection			
*Types of samples*	[[50], [51], [52], [53]]	Sample types that are appropriate for the research question should be collected.	The most accessible and commonly used sampling techniques (e.g. fecal samples) may not adequately capture the diet–microbiome interactions most relevant to host health outcomes.
*Quantity*	[48,50]	Will vary based on assay/measurement of interest.	Necessity of collecting complete sample and/or multiple samples per timepoint.
*Frequency*	[48,[50], [51], [52], [53]]	Multiple timepoints throughout study	Optimal number of samples per time point and number of time points to include are undetermined.
*Timing*	[[48], [49], [50],^52^,^53^]	Important to record and report time of collection.	Best approaches for incorporating collection timing, processing, and freezing in analyses are undetermined.
*Collection*	[48,52,53]	• Use standardized procedures • Home sampling with immediate freezing is best.	Universal agreement on standardized procedures for sample collection and transport when immediate freezing is not possible is needed.
*Transport, storage, and processing*	[48,50,52,53]	Maintain consistency in procedures throughout the study. Immediately freeze and store fecal samples at –80ºC for gut microbiome analysis.	
Fecal sample data to record	[[48], [49], [50],^53^]	Transit time should be measured and recorded.	• Impact of transit time on diet–gut microbiome interactions not fully characterized. • Whether indirect assessments of stool form (e.g. Bristol stool scale) are sufficient proxies for transit time and water content, and whether they can be used as a simple means of comparing dietary effects on fecal characteristics is unclear [49].
Microbiota analysis			
*Measurement methods*	[47,48,50,52,53]	Should be based on research questions and align with resolution needed to address study hypotheses, resource availability, and features of microbiome being studied.	No consensus on the best sequencing protocols.
*Relative vs. absolute abundance*	[[50], [51], [52], [53]]	None	• Availability of efficient and low-cost methods for measuring absolute abundance within the entire community is needed. • The extent to which the total number of bacteria in a community is important is unclear [49].
*Diversity measures*	[50,52,53]	Diversity metrics should be assessed and reported in combination with other measures of microbial community characteristics.	The essential diversity metrics to include in diet–microbiome studies have not been standardized.
*Reporting taxonomy*	[50,52]	Report database(s) and version used.	• Use of taxonomic assignment databases not standardized and can give different results. • Taxonomy classification and reporting are inconsistent, and the level of taxonomy required for understanding effects of diet–microbiome interactions on health outcomes is undefined.

#### Biological sample collection

Six publications recommended the best practices regarding various aspects of biological sample collection [[48], [49], [50], [51], [52], [53]] (see Table 3). Points of agreement included selecting sample types appropriate for the research question (2 of 3 publications; [52,53]), collecting sample quantities appropriate for the assay or measurement of interest (2 of 2 publications; [48, 50]), and collecting samples at several time points during a study (e.g. baseline, middle, endpoint, etc.) (3 publications; [[51], [52], [53]]). An additional point of agreement was to use standardized and consistent sample collection procedures throughout the study (3 of 5 publications; [48,50,52]). With respect to fecal sample collection specifically, points of agreement included immediately freezing and storing samples at –80ºC after collection (3 of 4 publications; [48,50,53]) and recording and reporting time of sample collection (4 of 4 publications; [[48], [49], [50],^53^]).

Several other recommendations were made by multiple studies but were not points of agreement among all studies commenting on a particular aspect of sample collection methodology. Two publications [48,50] recommended collecting multiple fecal samples per data collection period recommending collection periods of 2–3 d [50] and 3–7 d [48]. Two publications recommended that sample collections be aligned with dietary and biological assessments [48,52]. Two publications recommended minimizing freeze-thaw cycles and maintaining cold chain during sample collection and transport [50,53] whereas 3 publications noted that appropriate preservatives/additives can also be used in some situations [48,52,53] and can allow samples to be stored at ambient temperature for up to several weeks [48].

#### Fecal sample data to record

Four publications discussed the best practices for metadata to record when collecting fecal samples [[48], [49], [50],^53^]. Points of agreement were that transit time and time of sample collection should be measured and recorded (3 publications; [48,49,53]). If transit time cannot be directly measured, recording bowel movement frequency (2 publications; [48,50]) or Bristol stool scores (1 publication; [50]) were recommended as alternatives.

#### Microbiota analysis

##### Measurement methods

Five publications discussed the best practices for microbiota measurement methods [47,48, 50, 52,53]. A point of agreement was that the method chosen should be based on the research questions, resolution needed to address study hypotheses, resource availability, features of the microbiome being studied, dietary intervention, and response variables (4 publications; [[47], [48], 52,53]). Two publications recommended consistent methodology be used throughout an individual study [52,53]. Other recommendations from the reports were specific to high-throughput sequencing methods and included using nontemplate controls during library preparation to detect contaminant DNA (1 publication; [50]), controls to address batch effects (1 publication; [52]), and mock communities to identify potential sources of bias (1 publication; [53]).

##### Relative compared with absolute abundance

Four publications [[50], [51], [52], [53]] addressed the best practices regarding measuring and reporting of relative and absolute abundance. No points of agreement were identified. Two publications recommended measuring and reporting both relative and absolute measures, especially if DNA yields vary between samples [50,52]. Another publication recommended using qPCR in probiotic studies to aid with understanding probiotic persistence in the gut [51].

##### Diversity measures

Three publications [50,52,53] suggested the best practices for measuring diversity, focusing on metrics of microbial diversity specifically. A point of agreement across all 3 publications was that diversity metrics should be assessed in combination with other measures (e.g. structural stability over time). One publication also emphasized the importance of reporting the specific metric(s) used [52].

##### Reporting taxonomy

Best practices for reporting taxonomy were discussed in 2 publications [50,52]. Both agreed that the databases and versions used in bioinformatics pipelines should be reported.

### Analyzing, integrating and reporting diet and gut microbiome data

**FIGURE 5:**
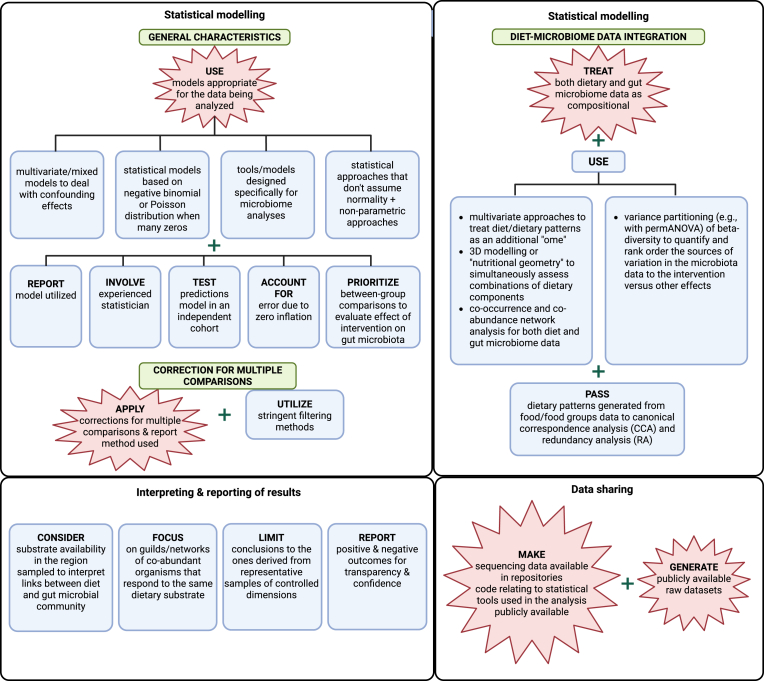
Synopsis of the best practice recommendations for analyzing, integrating, and reporting diet and gut microbiome data reported in diet–gut microbiome literature. Explosion boxes show points of agreement. Blue boxes show the best practice suggestions that were not points of agreement. Green boxes show the names of subelements. Arrows depict subelement leading or contributing to another subelement. Brackets show multiple best practice recommendations that apply to the same subelement. Created in BioRender. Diacova, T. (2025) https://BioRender.com/o91e099.

A summary of the results for the analyzing, integrating, and reporting diet and gut microbiome data category is provided in Table 4 [46,47,50,[52], [53]], Figure 5, and Supplemental Table 5. A summary of considerations and knowledge gaps is available in the Supplementary Results.

**TABLE 4:** Summary of points of agreement and knowledge gaps in diet–gut microbiome literature: analyzing, integrating, and reporting diet and gut microbiome data.

Category	References	Points of agreement	Gaps
Microbiome data transformation and normalization	[47,50,52,53]	Data should be transformed.	• Standardized best practices for choosing the most appropriate transformation and normalization methods are needed. • When log transformation is used the optimal pseudo count for zeros in the dataset is undetermined. • Appropriate use of rarefaction and other normalization strategies remains controversial.
Statistical modeling			
*General characteristics*	[46,47,50,52,53]	Use models appropriate for the data being analyzed	• Although the importance of utilizing gut microbiome specific models is recognized, there is no current consensus on the best statistical models for a given study design. • No consensus on the most appropriate way to deal with zero inflation or compositionality in datasets.
*Diet–microbiome data integration*	[46,52,53]	Both dietary and gut microbiome data are compositional and should be treated as such for analysis.	• Tools for treating dietary data as compositional and merging it with gut microbiome data for analysis are not well developed. • Most appropriate ways to apply ecological metrics to dietary data are unknown and multiple unanswered questions relating to implementing this approach and how to interpret results exist.
Correction for multiple comparisons	[47,50,52]	Correction for multiple comparisons should be applied and method reported.	None
Interpreting and reporting results	[50,52,53]	None	None
Data sharing	[50,52,53]	• Code relating to statistical tools used in the analysis should be made publicly available. • Raw datasets should be made available to the public. • Sequencing data should be made available in repositories.	None

#### Microbiome data transformation and normalization

Four publications [47,50,52,53] discussed the best practices for microbiome data transformation and normalization. A point of agreement among all 4 publications was that the data should be transformed in some way, but there was no agreement on the specific methodology to use. One publication stated that proportional and non-normally distributed data should be transformed [52]. Another suggested normalization and/or rarefaction for sequence count data depending on library size [47] and the remaining 2 publications mentioned the importance of rarefaction as well [50,53].

#### Statistical modeling

##### General characteristics

Five publications [46,47,50,52,53] discussed the best practices for statistical modeling in diet–gut microbiome research. A point of agreement among all publications was that models should be appropriate for the data being analyzed. Two publications further stated that models and tools designed specifically for microbiome analyses should be used [50,52]. One publication recommended using multivariate and mixed statistical models to deal with confounding effects [52], whereas another stressed the importance of nonparametric approaches [46]. One publication recommended accounting for error due to zero inflation (i.e. the presence of many zeros in a dataset) [46], with another recommending Poisson and negative binomial distributions to handle zero inflation [53].

##### Diet–microbiome data integration

Three publications [46,52,53] specifically addressed statistical approaches to diet–gut microbiota data integration. A point of agreement was that dietary data should be treated as compositional and as an additional “ome” and 3-dimensional modeling or “nutritional geometry” approaches are important to apply in analyses (2 publications; [46,52]).

#### Correction for multiple comparisons

Three publications [47,50,52] included the best practices for adjusting *P* values to reduce false-positives arising from statistical analyses on many related endpoints. A point of agreement across all 3 publications was that correction for multiple comparisons should be applied, and the method used reported. One publication also suggested utilizing stringent filtering methods when analyzing gut microbiome datasets to reduce type I error [52].

#### Reporting and interpreting results

Three publications [50,52,53] discussed the best practices for reporting and interpreting results in diet–gut microbiome studies. No points of agreement were identified, as each of the 3 publications addressed a different aspect of reporting. One publication highlighted the importance of discriminating biological and statistical significance while cautioning against overinterpreting results [50]. Another publication suggested focusing on networks of coabundant organisms that respond to the same dietary substrate and considering substrate availability and intestinal region sampled when interpreting results [52]. Finally, the third publication focused on the importance of reporting both positive and null outcomes for transparency and confidence [53].

#### Data sharing

Three publications [50,52,53] suggested the best practices for data sharing in diet–gut microbiome research. Points of agreement were that data (2 publications; [50,52]) and code relating to statistical tools used in the analysis (2 publications; [50,53]) be made publicly available.

## Discussion

Decisions required for conducting any human nutrition study include developing research questions and aims, identifying the target population, intervention, comparator and outcomes (PICO criteria), selecting the appropriate study design within that context, and constructing plans for administering interventions and measuring and analyzing study outcomes. Once the study is completed, methods and findings must be communicated clearly, transparently and in a way that facilitates reproducibility. Previous reports have discussed considerations that should inform that process [42,56,57]. In general, diet–gut microbiome studies should be designed, conducted, and reported based on the same principles but must also consider design categories that are unique to the study of diet–gut microbiome interactions. In this umbrella review, we consolidated current recommended best practices, considerations, and gaps in knowledge pertaining to the design, conduct, and reporting of research aiming to determine effects of diet–gut microbiome interactions on human health-related outcomes. Eight publications met all review criteria and were summarized. Notably, across all the categories and subcategories of study design and research methodology for which information was extracted, no category or subcategory was discussed by all 8 publications. Nonetheless, several points of agreement regarding the best practices were identified. Next, we discuss those best practices within the context of considerations and gaps extracted from the included publications and based on our own experience related to the design and conduct of studies aiming to identify the role of diet–gut microbiome interactions in human health.

### Study design and participant selection

Developing research questions and aims is integral to any human nutrition study. A challenge in diet–gut microbiome research highlighted by several of the included reviews is the difficulty in demonstrating that a specific interaction between a dietary component and the gut microbiome impacts health. For example, health effects of certain dietary fibers may be attributable to direct effects of that fiber in the gastrointestinal tract rather than, or in addition to, interactions with the gut microbiome. A diet–gut microbiome interaction could also impact human health through different pathways such as a microbiome-mediated biotransformation of a dietary component or through an impact of a dietary component on gut microbiome composition. Research questions and aims must therefore keep in mind both the nature of the diet–microbiome interaction and mechanism of action underlying health outcomes. As stated by Klurfeld et al. [49], if an aim is to convincingly demonstrate causal effects of a diet–gut microbiome interaction on human health, the inclusion of supporting mechanistic in vitro or animal studies needs to also be considered.

Across the publications reviewed, there was general recognition that RCTs, that use blinding when possible, are the best practice for determining effects of diet–gut microbiome interactions on health-related outcomes. However, there was no agreement on the most appropriate type of study design within the RCT framework. Crossover designs are appealing for diet–gut microbiome research because they can help control for the large interpersonal variability in the effects of diet on the gut microbiome that may depend, in part, on baseline gut microbiome composition [58]. Often, rapid baseline microbiome assessment, which could achieve the same goal in parallel arm studies by allowing participant stratification, is not feasible. On the other hand, crossover designs increase participant burden and may not be appropriate for outcomes that take a long time to develop. Furthermore, crossover designs require a washout period. Publications included in this review noted that 4 wk may be a sufficient washout duration to prevent carryover effects in diet–microbiome studies. However, we caution that the duration may vary based on the research question and intervention and may be unknown for many gut microbiome-targeted nutrition interventions. Additionally, if components of a dietary intervention are present in a participant’s usual diet, it may not always be possible to completely eliminate those components during the washout. Therefore, we suggest that the suitability of a particular RCT design in diet–gut microbiome research must ultimately consider the impact of interindividual variability along with the research question, study population, available resources, treatment duration, knowledge about the persistence of treatment effects, and outcome variables.

An additional factor that both influences and is influenced by the study design is sample size. Utilizing the “largest sample size possible” was suggested as the best practice in 1 publication [52] but, in our opinion, doing so may not be a time or resource-efficient approach to answer the research question. Several reviews agreed that physiological and biological endpoints and their biological/clinical significance should be considered when conducting effect size and sample size calculations. However, an identified challenge for sample size calculations in diet–gut microbiome studies is that the best way to measure microbiome responses to dietary interventions (e.g. taxa, metabolites or health markers) is not always clear and the gut microbiome features (if any) on which sample size calculations should be based are generally unknown.

Several publications noted that changes in gut microbial community composition and functionality can be used as indicators of response in some cases [46,53]. However, these cases may be limited given that the “healthy” gut microbiome remains undefined and widespread agreement on the compositional and/or functional changes having beneficial or detrimental effects on host health is lacking [59,60]. In support, several publications noted that high microbiome α-diversity (i.e. within-sample diversity) and gene richness are generally considered desirable in adults but do not equate to greater abundances of microbes performing beneficial functions and are not an absolute requirement for stability and resilience of the microbial ecosystem [50,52,53]. Indeed, some dietary interventions that produce beneficial health effects can reduce gut microbiome α-diversity [61]. For example, richness, a diversity metric that captures the total number of different taxa in a community, should not be expected to increase in response to a dietary intervention in which no exogenous microbes are added to the diet. Rather, certain health-promoting dietary components such as polyphenols may exert antibacterial effects that reduce community richness [62]. Health-promoting dietary interventions may also reduce α-diversity metrics of community evenness, reflecting both the number of taxa and their distribution in a community. One example is a dietary intervention that includes substrates utilized by many taxa, such as certain dietary fibers, which can reduce community evenness by skewing the distribution of the community toward highly abundant taxa that may out compete less abundant taxa for the new substrates [61]. In our opinion, further work on identifying gut microbiome biomarkers and related thresholds associated with health and disease risk is needed to better inform study designs in diet–gut microbiome research [59].

Defining gut microbiome biomarkers of health and disease would also help determine the optimal intervention durations for studies testing effects of microbiome-targeted nutrition interventions on host health. Papers included in this review noted that intervention durations required to demonstrate microbiome-mediated changes in host phenotype and to elicit long-term or permanent changes in the gut microbiome are generally unknown. Although 3–5 d may be sufficient duration for extreme dietary interventions to elicit changes in the gut microbiome [55], that duration is likely insufficient to meaningfully impact physiological outcomes, achieve gut microbiome stabilization or cause long-lasting impacts on the community. Identifying intervention durations required to sustain diet-mediated changes in the gut microbiome would help inform the designs of future diet–gut microbiome studies [63]. To do so, existing and future RCTs should consider incorporating follow-up assessments that allow for tracking the persistence of diet-mediated changes in the gut microbiome. However, in doing so, researchers should also consider that permanent change may not be possible without sustained intake of the dietary component that caused the microbiome to change. For example, frequent consumption of a selectively utilized dietary fiber may be necessary to support the survival of the bacterial species within a community capable of using that substrate.

Additional aspects of study design considered in this review were participant selection and important participant metadata in diet–gut microbiome research. Across the papers reviewed, there was general agreement on several factors that should be considered when establishing participant inclusion and exclusion criteria. Among those factors was the use of medications and -biotics likely to influence the gut microbiome, including antibiotics, prebiotics, and probiotics. Four weeks was identified as an appropriate washout period from different -biotics and establishing similar optimal washout durations for other relevant compounds such as medications would help in the standardization of diet–gut microbiome study designs. Multiple papers included in this review also suggested considering habitual diet and baseline gut microbiome [48,51,53] in study inclusion/exclusion criteria but specific guidance on how to best address these factors was lacking. Aside from biotic and medication use, habitual diet, and baseline gut microbiome, many other potential factors that could introduce variability into effects of a diet intervention on the gut microbiome were discussed. In our opinion, accounting for all possible factors in any study design or data analysis plan is generally not feasible (i.e. too many covariates result in overfit models). Therefore, we suggest that at minimum, diet–gut microbiome studies should report the critical metadata identified in this umbrella review and consider measuring, controlling, and/or reporting on other factors identified as potentially relevant covariates (Supplemental Table 2).

### Diet intervention and assessment

A notable finding of this review was that many gaps related to the development and measurement of nutritional interventions in diet–gut microbiome research exist. A common theme among the included publications was that multiple different factors within the diet (e.g. nutritive and non-nutritive compounds, food matrix and structure, preparation method, etc.) impact how a food, diet, or dietary component interacts with the gut microbiome. Reviewed publications agreed that developing nutrition-based interventions and appropriate controls should therefore consider those factors and attempt to reduce any potential impact on study results by matching interventions or diets and how they are consumed to the extent possible. That said, measuring and reporting on all potential factors that may impact how an intervention interacts with the gut microbiome is likely unachievable and not practical for most studies involving whole diets. Furthermore, the level of detail needed to ensure reproducibility for some outcomes is unclear. We suggest that developing a minimal reporting standard for various categories of nutrition-based interventions and background diets in diet–microbiome research would strengthen study designs and facilitate reproducibility. In the interim, we concur with Klurfeld et al. [49] who suggest that intervention descriptions should be as detailed as possible with respect to any provided items and the background diet.

Minimizing the effect of interindividual variability in habitual dietary intake was also identified as important to consider in diet–gut microbiome studies. However, there is currently no agreement within the field as to the best practices to achieve this. Two options discussed in the included reviews were diet standardization and diet stabilization. Standardizing intake by providing whole diets to study participants may help reduce the variability of the dietary effects on the microbiome that are being contributed by the basal diet. However, complete feeding studies are expensive, not feasible over very long time periods and can have limited external validity. The included publications varied in their descriptions of diet stabilization. Descriptions ranged from an approach to minimally disrupt participants’ habitual intake by instructing study participants to “maintain their standard diet” to a more laborious approach, such as designing a personalized menu so that each individual consumes the same foods/beverages over a prescribed amount of time before each sample collection period. We feel that it is important for the field to discuss and develop the best practice approaches to handling variability in dietary intake so that research methods can be harmonized. If diet stabilization is to be widely adopted in diet-gut microbiome research, we suggest that a standard definition of stabilization that is feasible to implement and verify is necessary.

Whether a diet is stabilized or standardized, an accurate measurement of the potential microbiome-modulating compounds in the diet is necessary. Unfortunately, accurate assessment of dietary intake is difficult [64]. The issue is not unique to diet–gut microbiome research and many efforts are underway to improve diet intake assessment [[65], [66], [67], [68], [69]]. Included publications agreed that assessment methods should allow for analysis of dietary patterns in addition to single nutrients if relevant to study aims. Included publications also noted that optimal methods for diet assessment in diet–gut microbiome studies may need to consider capturing details such as food temperature, which can be relevant to resistant starch content for example, and behaviors such as chewing, which can impact digestibility, and include recipe and cooking information. Included publications stated that timing and location of intake should also be recorded.

Once dietary intake is measured, databases are required to determine nutritional content. However, a major gap within diet–gut microbiome research is that no single database captures all information on dietary factors that may impact diet–microbiome interactions. This issue complicates comparisons across studies and can impact reproducibility. As stated by Johnson et al. [48], a shared food ontology that can harmonize dietary data collected in different global regions and by different tools may be one approach to addressing this gap. Adopting the emerging ecologic-based statistical approaches discussed by Choi et al. [46] may also help. Furthermore, more expansive nutrient databases that include specific nutritional components likely to affect the gut microbiome, such as different types of fibers (e.g. arabinoxylans, glucans) and phytochemicals, as well as live microbes, are necessary to help move the field forward. Though important and impactful efforts are underway in all these areas [[70], [71], [72], [73], [74], [75]], much additional work remains before complete and accessible databases with shared ontologies can be widely used.

Finally, the included publications agreed that measuring adherence to an intervention and any provided diet is a best practice. One identified method for measuring adherence while overcoming inaccuracies in diet measurement is measuring dietary intake biomarkers. Although there are few established biomarkers that can serve as objective biomarkers of dietary intake at present, work utilizing -omics technologies is showing promise for determining biomarkers of food intake and dietary patterns [[76], [77], [78], [79], [80], [81], [82], [83], [84]]. In our opinion, additional research to refine and expand dietary biomarkers, particularly those derived from diet–gut microbiome interactions, through novel methodologies is necessary to advance understanding of diet–microbiome relationships.

### Biological sample analysis

Included publications generally lacked agreement on concrete best practice recommendations for biological sample collection and analysis in diet–gut microbiome studies, providing nonspecific and in some cases inconsistent recommendations. Most diet–gut microbiome studies rely primarily on fecal sample collection for practical purposes. This can aid comparability across studies but, as noted by several of the included publications, may not fully capture diet–microbiome interactions most relevant to host health given that measurements in fecal samples differ from those taken in various sites along the gastrointestinal tract [85]. The continued development of noninvasive techniques for measuring diet–microbiome interactions throughout the gastrointestinal tract (e.g. [[85], [86], [87], [88]]) will help to advance understanding of those interactions in human health. Such devices may also help overcome some of the more practical limitations of fecal sample collection, which can include volunteer burden and hesitancy, and the inability to standardize collection timing.

Although included publications described sample type and quantity as dependent on the research question and outcomes of interest, there was general agreement that more frequent sample collections are better [48,[50], [51], [52], [53]]. Frequency, however, must be balanced with feasibility, which will depend, in part, on sample type (e.g. collection frequency will be limited in studies using biopsies). Ultimately, included publications did not agree on the optimal number of samples per time point and number of time points to include in diet–gut microbiome studies. In addition, although included publications agreed that transit time and time of sample collection are important metadata to collect, it was also noted that the impact of these factors on the gut microbiome and diet–gut microbiome interactions is not yet fully characterized.

Included publications also agreed that using consistent and standardized procedures for biological sample transport and storage within a study is a best practice. Unfortunately, standardizing procedures within a study does not necessarily facilitate between-study comparisons when different procedures are used in different studies. It is well recognized that results of studies relying on fecal samples can be influenced by how samples are collected, transported, and stored [21]. Likewise, differences in sample processing (e.g. DNA extraction method), sequencing protocols, and bioinformatics pipelines can introduce variability that may impact study results, their interpretation and comparison of results across studies [[89], [90], [91], [92], [93], [94], [95], [96], [97]]. These issues, combined with different collection and storage protocols, undoubtedly contribute to inconsistencies within the evidence base.

In general, there is a strong need for agreement regarding the best practices for sample collection, processing, and analysis and readers are referred to several reviews on the topic [[26], [27], [28], [29], [30], [38],^98^]. A critical and emerging area to address is how to best incorporate measures of absolute abundance into sequencing protocols that generate compositional data. A related question is whether full fecal samples or all samples produced over a given timeframe should be collected and the dry mass determined to better estimate total microbial abundances. In our opinion, establishing accessible best practices for measuring absolute abundances within the full community will facilitate measurement and reporting of changes in microbial cell counts which are likely to differ from changes in relative abundance [99]. Ultimately, the sample collection, processing, and analysis procedures used in any diet–gut microbiome research study will to some extent be based on research questions and providing the resolution needed to address study hypotheses within available resources and logistical constraints. However, we share the opinion of others [100] that improving the standardization of sample collection, processing, and analysis procedures will facilitate advancement and reproducibility in the field.

Finally, included reviews noted that the level of taxonomy required for understanding the effects of diet–gut microbiome interactions on health outcomes is undefined. Additionally, in our experience, the taxonomic levels and diversity metrics reported can differ across studies. These issues underlie the importance of reporting databases and versions used in bioinformatics pipelines and making raw data publicly available [[47], [48], [52]]. Doing so enables high-quality meta-analyses and improves reproducibility. However, developing standards for the taxonomic levels to report and diversity metrics to use would facilitate comparisons between studies. Given that most journals publish online supplementary tables and figures, we advocate for the approach suggested by Swann et al. [53] wherein results include multiple diversity metrics and all taxa included in differential abundance analyses so that both null and positive findings are transparently reported.

### Analyzing, integrating, and reporting diet and gut microbiome data

Many current diet–gut microbiome studies rely on 16S rRNA gene amplicon sequencing or, increasingly, shotgun sequencing. Both methods generate sparse (i.e. many zeros) and compositional (i.e. proportions) datasets that violate the assumptions of parametric statistical models. Thus, utilization of more advanced analytic approaches, including statistical models and tools that incorporate state-of-the-art approaches (e.g. [32,70,71,[101], [102], [103], [104], [105]]), may be best suited for analyzing diet–microbiome datasets and determining if and how the microbiome underpins physiological responses.

As with sample collection and analysis, the choice of statistical approach, from how the data are normalized and transformed to the statistical model used, can impact study results [101,103,[106], [107], [108], [109], [110]]. Despite promising and ongoing developments in statistical modeling for diet–gut microbiome research, significant gaps in knowledge exist. For example, the common use of rarefaction as a normalization method, which was endorsed by several of the included reviews, is a debated topic [111] and whereas half of the reviewed publications [47,50,52,53] indicated that compositional microbiome data should be transformed in some way, there was not agreement on the ideal transformation method(s). Furthermore, several included reviews mentioned the importance of using gut microbiome specific and multivariate models for statistical analysis of compositional data but there was not agreement on the best statistical models for a given study design or most appropriate ways to deal with zero inflation and compositionality. Standardizing analytical approaches for different types of microbiome data and study designs would facilitate between-study comparisons but is complicated by the frequent introduction of new, often improved modeling options. In our opinion, this will likely continue to complicate standardization in the field and underscores the imperative that researchers conducting diet–gut microbiome studies ensure their research teams remain current on data analysis methods. We suggest that, presently, one potential option for increasing confidence in study results is to compare results generated from multiple microbiome-specific statistical models and applying appropriate *P* value adjustments to reduce false-positive rates and control false discovery rates. Additionally, included reviews agreed that making data publicly available is a best practice [50,52-53]. Doing so facilitates reanalysis of existing data as improved methods continue to become available and can, in some cases, facilitate meta-analyses by allowing data from separate studies that were generated using the same microbiota measurement method (i.e. 16S rRNA amplicon sequencing, metagenomic sequencing) to be analyzed using the same bioinformatics and statistical analysis workflows. Of note, any meta-analyses of microbiome data should also consider biases introduced by different protocols, such as those resulting from different primer sets in 16S rRNA amplicon sequencing [112].

Like microbiome data, diet data can be considered compositional and treating it as such has potential to improve integration of these 2 data types. An emerging technique in the field is applying ecological approaches to dietary analysis to facilitate integration with compositional microbiome data [70,71,113]. Further development of these methods and others, including development of statistical techniques for mediation analysis that incorporate multiple highly multivariate model inputs, will likely facilitate new insights into diet–gut microbiome interactions and their impact on host health.

### Strengths and limitations

This umbrella review has several strengths and limitations that should be considered. Strengths include the use of standard methods to develop the review protocol [114] and preregistration. Furthermore, the narrow focus on publications related to design of studies assessing both diet and the gut microbiome allowed the review to capture recent discussions on the best practices unique to diet–gut microbiome research. However, a limitation to that narrow focus is we did not extract information from publications focused solely on the design of microbiome or nutrition studies, respectively. Thus, the extracted information may not fully capture all relevant best practices and considerations for conducting microbiome or nutrition studies per se. An additional limitation is that only one database was searched and relevant publications could have been missed. The search results, however, were supplemented with the manual screening of reference lists from included publications and other related reviews as well as coauthor input. This review only included publications that focused on adult and free-living populations and therefore may not capture unique aspects of study design and research methodology for other populations, such as infants or those with chronic diseases. Finally, this review only included papers published in the past 10 y (i.e. 2013–2023). That criterion may have excluded relevant literature but likely also minimized the inclusion of obsolete recommendations and considerations as nutritional microbiology is a rapidly evolving field.

In conclusion, a recurring theme of the included publications was that the best practices will depend on the research question, specific aims, outcomes, and feasibility of the study. Although true, we suggest that addressing several identified gaps will help facilitate establishing broadly applicable best practices for RCTs focused on diet–gut microbiome interactions in human health that are ultimately needed to improve reproducibility and advance the field. Several of those gaps are not unique to diet–microbiome research. These include issues that challenge nutrition science in general such as difficulty in accurately assessing dietary intake, an absence of validated intake biomarkers for many nutrients and diet components, and incomplete food and nutrient databases. Other gaps relevant across the field of gut microbiology are largely related to standardizing the best practices for sample collection, processing, transport and storage, sequencing protocols, bioinformatics workflows, and optimal statistical approaches for dealing with sparse and compositional data. Also, not unique to diet–gut microbiome research is the importance of developing devices for in situ measurement within the gastrointestinal tract, accounting for interindividual variability across human gut microbiomes and a need to define a healthy microbiome, if possible. The latter would assist in identifying meaningful biomarkers, effects, and effect sizes. Gaps more unique to the study of diet–gut microbiome interactions in human health include improving and harmonizing existing food and nutrient databases to account for currently underrepresented microbiome-modulating dietary components; determining the dietary components that are absolutely critical to match between nutrition interventions and controls; improving understanding of intervention durations required to elicit durable changes in the gut microbiome; and further developing and refining statistical models for integrating diet and gut microbiome data.

Addressing these gaps will require continued efforts from multidisciplinary research groups and many gaps will take years to resolve. However, resolution can be facilitated by cross-disciplinary exchange on specific priority areas where enough progress might be made to reach agreement within the nutritional microbiology community in the near term. We suggest that potential priority areas could include:

1) Developing reporting standards for diets and dietary interventions in diet–gut microbiome studies. These would build on previous recommendations [49] and be published with a checklist. The reporting standards could also be expanded to include metrics for microbiome-related outcomes (e.g. diversity metrics, summary statistics, etc.) and be designed to complement existing reporting recommendations for microbiome studies [100].
2) Identifying key gut–microbiome modulating dietary components to incorporate into commonly used nutrition databases including fiber types, polyphenolic compounds, non-nutritive compounds (e.g. artificial sweeteners, emulsifiers, etc.), and foodborne microbes. The dietary components identified would also inform guidelines for designing study interventions and diets.
3) Identifying gut microbiome features or gut microbiome-derived compounds and associated thresholds that can or are candidates to serve as health biomarkers. Relatedly, continuing discussion of gut microbiome features that may be characteristic of a “healthy” gut microbiome.
4) Developing guidelines for biological sample collection, processing, and analysis for key endpoints in diet–gut microbiome studies to include standardized methods for assessing microbiome composition and function and biomarkers of diet–gut microbiome interactions (e.g. short-chain fatty acids). Guidelines should consider how to minimize the impact of factors that may influence study results such as transit time, time of sample collection, amount of sample collected (e.g. small portion, whole sample or multiple consecutive whole samples for fecal samples), and variations in bioinformatics workflows.

Though future research and expert discussions are needed to address these gaps and establish and evolve the best practices in the field, significant efforts are already underway. As those efforts continue, the best practices and considerations identified in this review, the publications from which information was extracted and other relevant publications cited throughout can provide a guide for designing and conducting diet–gut microbiome studies. Research teams leading those efforts should strive to be multidisciplinary to ensure existing and new best practices can be adopted and implemented. Study designs should be based on research questions and aims and be adequately powered with physiologically meaningful endpoints. Gut microbiome-modulating dietary factors should be carefully considered in developing interventions and controls, whereas sample collection and analysis protocols should be consistent with study aims and use standards when available. Data should be analyzed with appropriate statistical models. Studies should also be reported transparently following guidelines for reporting clinical trials [115] and microbiome studies [100]. Finally, to the extent possible, study data, especially sequencing and dietary intake data, should be made publicly available to facilitate reanalysis and high-quality meta-analysis as more evidence for various interventions and endpoints becomes available and as databases and methods continue to improve.

## Author contributions

The authors’ responsibilities were as follows – TD, JPK: conducted the research and wrote the paper; JPK: primary responsibility for final content; and all authors: edited the manuscript, designed the research, and read and approved the final manuscript.

## Data availability

Data described in the manuscript will be made publicly and freely available without restriction at https://doi.org/10.17605/OSF.IO/FUPZS.

## Disclaimer

The opinions or assertions contained herein are the private views of the authors and are not to be construed as official or reflecting the views of the Army or the Department of Defense. Any citations of commercial organizations and trade names in this report do not constitute an official Department of the Army endorsement or approval of the products or services of these organizations. Approved for public release; distribution is unlimited.

## Funding

The authors reported no funding received for this study.

## Conflict of interest

The group that conducted this work was organized by the Institute for the Advancement of Food and Nutrition Sciences (IAFNS). IAFNS is a nonprofit science organization that pools funding from industry and advances science through the in-kind and financial contributions from private and public sector members. Authors received no funding from IAFNS to conduct this work. Authors have no conflicts of interest related to this work to disclose. Cindy Davis is an Editorial Board Member for Advances in Nutrition and played no role in the journal’s evaluation of the manuscript.

## References

[1] Rinninella E., Tohumcu E., Raoul P., Fiorani M., Cintoni M., Mele M.C. (2023). The role of diet in shaping human gut microbiota. Best Pract. Res. Clin. Gastroenterol..

[2] Ansaldo E., Farley T.K., Belkaid Y. (2021). Control of immunity by the microbiota. Annu. Rev. Immunol..

[3] Ghosh S., Whitley C.S., Haribabu B., Jala V.R. (2021). Regulation of intestinal barrier function by microbial metabolites. Cell Mol. Gastroenterol. Hepatol..

[4] Morais L.H., Schreiber H.L., Mazmanian S.K. (2021). The gut microbiota–brain axis in behaviour and brain disorders. Nat. Rev. Microbiol..

[5] Chen Y., Zhou J., Wang L. (2021). Role and mechanism of gut microbiota in human disease. Front Cell Infect. Microbiol..

[6] Gomaa E.Z. (2020). Human gut microbiota/microbiome in health and diseases: a review. Antonie van Leeuwenhoek.

[7] Illiano P., Brambilla R., Parolini C. (2020). The mutual interplay of gut microbiota, diet and human disease. FEBS J.

[8] Afzaal M., Saeed F., Shah Y.A., Hussain M., Rabail R., Socol C.T. (2022). Human gut microbiota in health and disease: unveiling the relationship. Front. Microbiol..

[9] Nesci A., Carnuccio C., Ruggieri V., D’Alessandro A., Di Giorgio A., Santoro L. (2023). Gut microbiota and cardiovascular disease: evidence on the metabolic and inflammatory background of a complex relationship. Int. J. Mol. Sci..

[10] Fan Y., Pedersen O. (2021). Gut microbiota in human metabolic health and disease. Nat. Rev. Microbiol..

[11] Ramos S., Martín M.Á. (2021). Impact of diet on gut microbiota. Curr. Opin. Food Sci..

[12] Armet A.M., Deehan E.C., O'Sullivan A.F., Mota J.F., Field C.J., Prado C.M. (2022). Rethinking healthy eating in light of the gut microbiome. Cell Host Microbe.

[13] Li C. (2023). Understanding interactions among diet, host and gut microbiota for personalized nutrition. Life Sci.

[14] Wolter M., Grant E.T., Boudaud M., Steimle A., Pereira G.V., Martens E.C. (2021). Leveraging diet to engineer the gut microbiome. Nat. Rev. Gastroenterol. Hepatol..

[15] Rahman S., O'Connor A.L., Becker S.L., Patel R.K., Martindale R.G., Tsikitis V.L. (2023). Gut microbial metabolites and its impact on human health. Ann. Gastroenterol..

[16] Dasriya V.L., Samtiya M., Ranveer S., Dhillon H.S., Devi N., Sharma V. (2024). Modulation of gut-microbiota through probiotics and dietary interventions to improve host health. J. Sci. Food Agric..

[17] Rothschild D., Weissbrod O., Barkan E., Kurilshikov A., Korem T., Zeevi D. (2018). Environment dominates over host genetics in shaping human gut microbiota. Nature.

[18] Ross F.C., Patanglia D., Grimaud G., Lavalle A., Dempsey E.M., Ross R.P., Stanton C. (2024). The interplay between diet and the gut microbiome: implications for health and disease. Nat Rev Microbiol.

[19] Vernocchi P., Del Chierico F., Putignani L. (2020). Gut microbiota metabolism and interaction with food components. Int. J. Mol. Sci..

[20] Thomson C., Garcia A.L., Edwards C.A. (2021). Interactions between dietary fibre and the gut microbiota. Proc. Nutr. Soc..

[21] Wu S., Bhat Z.F., Gounder R.S., Mohamed Ahmed I.A., Al-Juhaimi F.Y., Ding Y. (2022). Effect of dietary protein and processing on gut microbiota - a systematic review. Nutrients.

[22] Peled S., Livney YD. (2021). The role of dietary proteins and carbohydrates in gut microbiome composition and activity: a review. Food Hydrocoll.

[23] Koh A., Bäckhed F. (2020). From association to causality: the role of the gut microbiota and its functional products on host metabolism. Mol. Cell..

[24] Gagnon E., Mitchell P.L., Manikpurage H.D., Abner E., Taba N., Esko T. (2023). Impact of the gut microbiota and associated metabolites on cardiometabolic traits, chronic diseases and human longevity: a Mendelian randomization study. J. Transl. Med..

[25] Sanna S., Kurilshikov A., van der Graaf A., Fu J., Zhernakova A. (2022). Challenges and future directions for studying effects of host genetics on the gut microbiome. Nat. Genet..

[26] Fraher M.H., O’Toole P.W., Quigley E.M. (2012). Techniques used to characterize the gut microbiota: a guide for the clinician. Nat. Rev. Gastroenterol. Hepatol..

[27] Allaband C., McDonald D., Vázquez-Baeza Y., Minich J.J., Tripathi A., Brenner D.A. (2019). Microbiome 101: studying, analyzing, and interpreting gut microbiome data for clinicians. Clin. Gastroenterol. Hepatol..

[28] Maki K.A., Diallo A.F., Lockwood M.B., Franks A.T., Green S.J., Joseph P.V. (2019). Considerations when designing a microbiome study: implications for nursing science. Biol. Res. Nurs..

[29] Poussin C., Sierro N., Boué S., Battey J., Scotti E., Belcastro V. (2018). Interrogating the microbiome: experimental and computational considerations in support of study reproducibility. Drug Discov. Today..

[30] Qian X.B., Chen T., Xu Y.P., Chen L., Sun F.X., Lu M.P. (2020). A guide to human microbiome research: study design, sample collection, and bioinformatics analysis. Chin. Med. J. (Engl)..

[31] Claesson M.J., Clooney A.G., O’Toole P.W. (2017). A clinician's guide to microbiome analysis. Nat. Rev. Gastroenterol. Hepatol..

[32] Jiang D., Armour C.R., Hu C., Mei M., Tian C., Sharpton T.J. (2019). Microbiome multi-omics network analysis: statistical considerations, limitations, and opportunities. Front. Genet..

[33] Zhang X., Figeys D. (2019). Perspective and guidelines for metaproteomics in microbiome studies. J. Proteom. Res..

[34] Gheorghe C.E., Ritz N.L., Martin J.A., Wardill H.R., Cryan J.F., Clarke G. (2021). Investigating causality with fecal microbiota transplantation in rodents: applications, recommendations and pitfalls. Gut. Microbes..

[35] Debelius J., Song S.J., Vazquez-Baeza Y., Xu Z.Z., Gonzalez A., Knight R. (2016). Tiny microbes, enormous impacts: what matters in gut microbiome studies?. Genome Biol.

[36] Issa Isaac N., Philippe D., Nicholas A., Raoult D., Eric C. (2019). Metaproteomics of the human gut microbiota: challenges and contributions to other OMICS. Clin. Mass Spectrom.

[37] Shah R.M., McKenzie E.J., Rosin M.T., Jadhav S.R., Gondalia S.V., Rosendale D. (2020). An integrated multi-disciplinary perspective for addressing challenges of the human gut microbiome. Metabolites.

[38] de la Cuesta-Zuluaga J., Escobar J.S. (2016). Considerations for optimizing microbiome analysis using a marker gene. Front. Nutr..

[39] Relman D.A. (2020). Thinking about the microbiome as a causal factor in human health and disease: philosophical and experimental considerations. Curr. Opin. Microbiol..

[40] Casals-Pascual C., González A., Vázquez-Baeza Y., Song S.J., Jiang L., Knight R. (2020). Microbial diversity in clinical microbiome studies: sample size and statistical power considerations. Gastroenterology.

[41] Liu Y.X., Qin Y., Chen T., Lu M., Qian X., Guo X. (2021). A practical guide to amplicon and metagenomic analysis of microbiome data. Protein Cell.

[42] Lichtenstein A.H., Petersen K., Barger K., Hansen K.E., Anderson C.A., Baer D.J. (2021). Perspective: design and conduct of human nutrition randomized controlled trials. Adv. Nutr..

[43] Weaver C.M., Lichtenstein A.H., Kris-Etherton P.M. (2021). Perspective: guidelines needed for the conduct of human nutrition randomized controlled trials. Adv. Nutr..

[44] Weaver C.M., Fukagawa N.K., Liska D., Mattes R.D., Matuszek G., Nieves J.W. (2021). Perspective: US documentation and regulation of human nutrition randomized controlled trials. Adv. Nutr..

[45] Maki K.C., Miller J.W., McCabe G.P., Raman G., Kris-Etherton P.M. (2021). Perspective: laboratory considerations and clinical data management for human nutrition randomized controlled trials: guidance for ensuring quality and integrity. Adv. Nutr..

[46] Choi Y., Hoops S.L., Thoma C.J., Johnson A.J. (2022). A guide to dietary pattern-microbiome data integration. J. Nutr..

[47] Hughes R.L., Marco M.L., Hughes J.P., Keim N.L., Kable M.E. (2019). The role of the gut microbiome in predicting response to diet and the development of precision nutrition models – part I: overview of current methods. Adv. Nutr..

[48] Johnson A.J., Zheng J.J., Kang J.W., Saboe A., Knights D., Zivkovic A.M. (2020). A guide to diet-microbiome study design. Front. Nutr..

[49] Klurfeld D.M., Davis C.D., Karp R.W., Allen-Vercoe E., Chang E.B., Chassaing B. (2018). Considerations for best practices in studies of fiber or other dietary components and the intestinal microbiome. Am. J. Physiol. Endocrinol. Metab..

[50] Marques F.Z., Jama H.A., Tsyganov K., Gill P.A., Rhys-Jones D., Muralitharan R.R. (2019). Guidelines for transparency on gut microbiome studies in essential and experimental hypertension. Hypertension.

[51] Mohr A.E., Pugh J., O’Sullivan O., Black K., Townsend J.R., Pyne D.B. (2022). Best practices for probiotic research in athletic and physically active populations: guidance for future randomized controlled trials. Front. Nutr..

[52] Shanahan E.R., McMaster J.J., Staudacher H.M. (2021). Conducting research on diet-microbiome interactions: a review of current challenges, essential methodological principles, and recommendations for best practice in study design. J. Hum. Nut. Diet..

[53] Swann J.R., Rajilic-Stojanovic M., Salonen A., Sakwinska O., Gill C., Meynier A. (2020). Considerations for the design and conduct of human gut microbiota intervention studies relating to foods. Eur. J. Nutr..

[54] (2023). Abstrackr. http://abstrackr.cebm.brown.edu/.

[55] David L.A., Maurice C.F., Carmody R.N., Gootenberg D.B., Button J.E., Wolfe B.E. (2014). Diet rapidly and reproducibly alters the human gut microbiome. Nature.

[56] Sackett D.L. (1997). Evidence-based medicine. Semin. Perinatol..

[57] Browner W.S. (2012).

[58] Healey G.R., Murphy R., Brough L., Butts C.A., Coad J. (2017). Interindividual variability in gut microbiota and host response to dietary interventions. Nutr. Rev..

[59] Verbeke K.A., Boobis A.R., Chiodini A., Edwards C.A., Franck A., Kleerebezem M. (2015). Towards microbial fermentation metabolites as markers for health benefits of prebiotics. Nut. Res. Rev..

[60] McBurney M.I., Davis C., Fraser C.M., Schneeman B.O., Huttenhower C., Verbeke K. (2019). Establishing what constitutes a healthy human gut microbiome: state of the science, regulatory considerations, and future directions. J. Nutr..

[61] Cantu-Jungles T.M., Hamaker B.R. (2023). Tuning expectations to reality: don't expect increased gut microbiota diversity with dietary fiber. J. Nutr..

[62] Makarewicz M., Drożdż I., Tarko T., Duda-Chodak A. (2021). The interactions between polyphenols and microorganisms. especially gut microbiota, Antioxidants (Basel).

[63] Leeming E.R., Johnson A.J., Spector T.D., Le Roy C.I. (2019). Effect of diet on the gut microbiota: rethinking intervention duration. Nutrients.

[64] Kirkpatrick S.I., Baranowski T., Subar A.F., Tooze J.A., Frongillo E.A. (2019). Best practices for conducting and interpreting studies to validate self-report dietary assessment methods. J. Acad. Nutr. Diet..

[65] Larke J.A., Chin E.L., Bouzid Y.Y., Nguyen T., Vainberg Y., Lee D.H. (2023). Surveying Nutrient Assessment with Photographs of Meals (SNAPMe): a benchmark dataset of food photos for dietary assessment. Nutrients.

[66] Shonkoff E., Cara K.C., Pei X.A., Chung M., Kamath S., Panetta K. (2023). AI-based digital image dietary assessment methods compared to humans and ground truth: a systematic review. Ann. Med..

[67] Bromage S., Fung T.T., Kirkpatrick S.I., Willett W.C. (2023). A video repository for innovative methods of dietary assessment and analysis. J. Vis. Exp..

[68] Vadiveloo M.K., Juul F., Sotos-Prieto M., Parekh N. (2022). Perspective: novel approaches to evaluate dietary quality: combining methods to enhance measurement for dietary surveillance and interventions. Adv. Nutr..

[69] Bernstein A.M., Rhee L.Q., Njike V.Y., Katz D.L. (2023). Dietary assessment by pattern recognition: a comparative analysis. Curr. Dev. Nutr..

[70] Sadohara R., Johnson A. (2023). P29-039-23 DietR-A dietary analysis tool for ASA24 and NHANES dietary data in R, Curr. Dev. Nutr..

[71] Sadohara R., Jacobs D., Pereira M.A., Johnson A.J. (2023). Dietary pattern and diversity analysis using ‘DietR’package in R. medRxiv.

[72] Kay C.D., Clifford M.N., Mena P., McDougall G.J., Andres-Lacueva C., Cassidy A. (2020). Recommendations for standardizing nomenclature for dietary (poly)phenol catabolites. Am. J. Clin. Nutr..

[73] (2019). FoodB [Internet].

[74] Dooley D.M., Griffiths E.J., Gosal G.S., Buttigieg P.L., Hoehndorf R., Lange M.C. (2018). FoodOn: a harmonized food ontology to increase global food traceability, quality control and data integration. NPJ Sci. Food..

[75] Rothwell J.A., Pérez-Jiménez J., Neveu V., Medina-Remón A., M’Hiri N., Garcia Lobato P. (2013). Phenol-Explorer 3.0: a major update of the Phenol-Explorer database to incorporate data on the effects of food processing on polyphenol content. Database (Oxford).

[76] Maruvada P., Lampe J.W., Wishart D.S., Barupal D., Chester D.N., Dodd D. (2020). Perspective: dietary biomarkers of intake and exposure - exploration with omics approaches. Adv. Nutr..

[77] Vázquez-Manjarrez N., Weinert C.H., Ulaszewska M.M., Mack C.I., Micheau P., Pétéra M. (2019). Discovery and validation of banana intake biomarkers using untargeted metabolomics in human intervention and cross-sectional studies. J. Nutr..

[78] Shinn L.M., Mansharamani A., Baer D.J., Novotny J.A., Charron C.S., Khan N.A. (2023). Fecal metabolites as biomarkers for predicting food intake by healthy adults. J. Nutr..

[79] D'Angelo S., Gormley I.C., McNamara A.E., Brennan L. (2021). multiMarker: software for modelling and prediction of continuous food intake using multiple biomarkers measurements. BMC Bioinformatics.

[80] Li K.J., Burton-Pimentel K.J., Brouwer-Brolsma E.M., Blaser C., Badertscher R., Pimentel G. (2023). Identifying plasma and urinary biomarkers of fermented food intake and their associations with cardiometabolic health in a Dutch observational cohort. J. Agric. Food Chem..

[81] Tang Y., Zhu Y., Sang S. (2020). A novel LC-MS based targeted metabolomic approach to study the biomarkers of food intake. Mol. Nutr. Food Res..

[82] Garcia-Aloy M., Ulaszewska M., Franceschi P., Estruel-Amades S., Weinert C.H., Tor-Roca A. (2020). Discovery of intake biomarkers of lentils, chickpeas, and white beans by untargeted LC-MS metabolomics in serum and urine. Mol. Nutr. Food Res..

[83] Levy J., Silva A.M., De Carli E., Cacau L.T., de Alvarenga J.F.R., Fiamoncini J. (2022). Biomarkers of fruit intake using a targeted metabolomics approach: an observational cross-sectional analysis of the ELSA-Brasil Study. J. Nutr..

[84] Liang S., Nasir R.F., Bell-Anderson K.S., Toniutti C.A., O'Leary F.M., Skilton M.R. (2022). Biomarkers of dietary patterns: a systematic review of randomized controlled trials. Nutr. Rev.

[85] Shalon D., Culver R.N., Grembi J.A., Folz J., Treit P.V., Shi H. (2023). Profiling the human intestinal environment under physiological conditions. Nature.

[86] Kalantar-Zadeh K., Yao C.K., Berean K.J., Ha N., Ou J.Z., Ward S.A. (2016). Intestinal gas capsules: a proof-of-concept demonstration. Gastroenterology.

[87] Mimee M., Nadeau P., Hayward A., Carim S., Flanagan S., Jerger L. (2018). An ingestible bacterial-electronic system to monitor gastrointestinal health. Science.

[88] Thwaites P.A., Yao C.K., Halmos E.P., Muir J.G., Burgell R.E., Berean K.J. (2024). Review article: current status and future directions of ingestible electronic devices in gastroenterology, Aliment. Pharmacol. Ther..

[89] Wang Z., Zolnik C.P., Qiu Y., Usyk M., Wang T., Strickler H.D. (2018). Comparison of fecal collection methods for microbiome and metabolomics studies. Front. Cell Infect. Microbiol..

[90] Maghini D.G., Dvorak M., Dahlen A., Roos M., Kuersten S., Bhatt A.S. (2024). Quantifying bias introduced by sample collection in relative and absolute microbiome measurements. Nat. Biotechnol..

[91] Vandeputte D., Tito R.Y., Vanleeuwen R., Falony G., Raes J. (2017). Practical considerations for large-scale gut microbiome studies. FEMS Microbiol. Rev..

[92] Vogtmann E., Chen J., Amir A., Shi J., Abnet C.C., Nelson H. (2017). Comparison of collection methods for fecal samples in microbiome studies. Am. J. Epidemiol..

[93] Sergaki C., Anwar S., Fritzsche M., Mate R., Francis R.J., MacLellan-Gibson K. (2022). Developing whole cell standards for the microbiome field. Microbiome.

[94] Amos G.C., Logan A., Anwar S., Fritzsche M., Mate R., Bleazard T. (2020). Developing standards for the microbiome field. Microbiome.

[95] Pérez-Cobas A.E., Gomez-Valero L., Buchrieser C. (2020). Metagenomic approaches in microbial ecology: an update on whole-genome and marker gene sequencing analyses. Microb. Genom..

[96] Callahan B.J., McMurdie P.J., Holmes S.P. (2017). Exact sequence variants should replace operational taxonomic units in marker-gene data analysis. ISME J.

[97] Costea P.I., Zeller G., Sunagawa S., Pelletier E., Alberti A., Levenez F. (2017). Towards standards for human fecal sample processing in metagenomic studies. Nat. Biotechnol..

[98] Knight R., Vrbanac A., Taylor B.C., Aksenov A., Callewaert C., Debelius J. (2018). Best practices for analysing microbiomes. Nat. Rev. Microbiol..

[99] Wang X., Howe S., Deng F., Zhao J. (2021). Current aplications of absolute bacterial quantification in microbiome and decision-making regarding different biological questions. Microorganisms.

[100] Mirzayi C., Renson A., Zohra F., Elsafoury S. (2021).

[101] Mallick H., Rahnavard A., McIver L.J., Ma S., Zhang Y., Nguyen L.H. (2021). Multivariable association discovery in population-scale meta-omics studies. PLOS Comput. Biol..

[102] Guo B., Holscher H.D., Auvil L.S., Welge M.E., Bushell C.B., Novotny J.A. (2023). Estimating heterogeneous treatment effect on multivariate responses using random forests. Stat. Biosci..

[103] Gloor G.B., Macklaim J.M., Pawlowsky-Glahn V., Egozcue J.J. (2017). Microbiome datasets are compositional: and this is not optional. Front. Microbiol..

[104] Gibson T., Gerber G. (2018). Proceedings of the 35th International Conference on Machine Learning; 2018 Jul 10–15.

[105] Chong J., Xia J. (2017). Computational approaches for integrative analysis of the metabolome and microbiome. Metabolites.

[106] McMurdie P.J., Holmes S. (2014). Waste not, want not: why rarefying microbiome data is inadmissible. PLOS Comput. Biol..

[107] Lloréns-Rico V., Vieira-Silva S., Gonçalves P.J., Falony G., Raes J. (2021). Benchmarking microbiome transformations favors experimental quantitative approaches to address compositionality and sampling depth biases. Nat. Commun..

[108] Wright R.J., Comeau A.M., Langille M.G. (2023). From defaults to databases: parameter and database choice dramatically impact the performance of metagenomic taxonomic classification tools. Microb. Genom..

[109] Bharti R., Grimm D.G. (2021). Current challenges and best-practice protocols for microbiome analysis. Brief Bioinform.

[110] Weiss S., Xu Z.Z., Peddada S., Amir A., Bittinger K., Gonzalez A. (2017). Normalization and microbial differential abundance strategies depend upon data characteristics. Microbiome.

[111] Schloss P.D. (2024). Waste not, want not: revisiting the analysis that called into question the practice of rarefaction. mSphere.

[112] Lozupone C.A., Stombaugh J., Gonzalez A., Ackermann G., Wendel D., Vázquez-Baeza Y. (2013). Meta-analyses of studies of the human microbiota. Genome Res.

[113] Eetemadi A., Rai N., Pereira B.M., Kim M., Schmitz H., Tagkopoulos I. (2020). The computational diet: a review of computational methods across diet, microbiome, and health. Front. Microbiol..

[114] Aromataris E., Fernandez R., Godfrey C., Holly C., Khalil H., Tungpunkom P., Aromataris E., Lockwood C., Porritt K., Pilla B., Jordan Z. (2024). JBI Manual for Evidence Synthesis.

[115] Cuschieri S. (2019). The CONSORT statement. Saudi J. Anaesth..

