# Supplementary material for: Best Practices and Considerations for Conducting Research on Diet–Gut Microbiome Interactions and Their Impact on Health in Adult Populations: An Umbrella Review
Source: Adv Nutr. 2025 Apr 1;16(5):100419. doi: 10.1016/j.advnut.2025.100419 (PMC12056254; doi:10.1016/j.advnut.2025.100419)
Supplement: Multimedia component 1 [file mmc1.pdf]

Diacova et al.; Best Practices and Considerations for Conducting Research on Diet-Gut Microbiome Interactions and their Impact on Health in Adult Populations: An Umbrella Review

Supplementary Materials

Supplementary Results

The text below summarizes considerations and knowledge gaps extracted from the eight publications (1-8) included in this umbrella review.

1. Study design and participant selection

A summary of the results for the study design and participant selection category is provided in **Table 1** and **Figure 2** of the main text and **Supplementary Table 2**. Below we summarize the extracted considerations and knowledge gaps.

1.1 Research questions and aims

One publication (8) discussed considerations for research questions and aims, describing several broad, common research questions often considered in diet-gut microbiome studies. These questions included the effects of the gut microbiome on metabolism and bioavailability of nutrients and non-nutrient foods, how that metabolism influences human health, the effects of diet, food or food components on the gut microbiome composition and function, and the effects of gut microbiome changes on human health and biomarkers of health. The authors also noted that some health-related changes may be occurring in parallel but independently of shifts in the gut microbiome (8), which can complicate causal inference and therefore should be considered when developing research questions and aims.

1.2 Trial design and blinding

Four publications (4, 6-8) discussed considerations for trial design and blinding:

Crossover designs were discussed as having several advantages for diet-gut microbiome studies. Those advantages were described as providing the potential to test dose-response relationships and controlling confounding from habitual diet and baseline microbial communities that often demonstrate large inter-individual variability (7). Mohr et al. (6) agreed that when responses are thought to strongly be driven by the underlying gut microbiome a crossover design is optimal. Klurfeld et al. (4), however, pointed out that crossover studies usually are limited to relatively short-term interventions, increase volunteer burden, and prevent evaluating hard disease end points. **Parallel designs** were described as less burdensome than crossover trials on volunteers and potentially better suited for certain diet-gut microbiome studies where longer-term interventions, concerns of attrition, and lengthy washout periods preclude using a crossover design (6). **Adaptive trials** (9), which can be applied to both crossover and parallel studies, were identified as another trial design option to consider in diet gut-microbiome research (6). Mohr et al. (6) explained that within an adaptive design, planning to be flexible is encouraged, interim data examination is allowed without compromising the integrity of the trial, and follow-up assessments are encouraged to obtain information on dissipation of effects and persistence of interventions.

An additional consideration discussed within the reviewed publications is that the longitudinal nature of both crossover and parallel studies allows for measuring the effect of temporal dietary variation and the effect of an intervention on microbial composition and stability within an individual (7). Design of those studies can often be improved when informed by observational and exploratory or pilot research that facilitates the generation of testable hypotheses (7) and provides information necessary to design and adequately power clinical trials. Swann et al. (8) described **exploratory and pilot trials** as particularly useful in diet-gut microbiome

research as optimal intervention doses, persistence of any effect, variability and effect sizes are often unknown but integral for study design.

A final consideration discussed by Klurfeld et al. (4) is that demonstrating that a diet-gut microbiome interaction causes a particular health effect in humans is extremely challenging. Doing so and identifying the underpinning mechanisms often requires a combination of well-designed human and animal studies.

1.3 Duration of run-in, washout, and intervention

Three publications (3-4, 6) discussed considerations for the duration of run-in, washout, and intervention periods. Mohr et al. (6) stated that **run-in periods** may be beneficial depending on the research questions and study design, and to gauge intervention compliance and ability to complete outcome measures. **Intervention duration** will vary based on the research question and primary outcomes. Notably, the duration required to demonstrate microbiome-mediated changes in host phenotype is likely on the order of weeks to months (3). In carryover studies, **washout** periods between treatment arms are required and should be of sufficient duration to prevent carryover effects (3). Mohr et al. (6) noted that for some interventions such as probiotics, a minimum washout period of 2-3 weeks should be implemented. Other reports stated that the washout periods should be at least 2 weeks (4) and 4 weeks (5). For parallel group studies, one report encouraged follow-up assessments to determine the extent to which any intervention-mediated changes in the gut microbiome persist long-term (6), which is also useful for informing appropriate washout periods for future crossover studies.

1.4 Effect size and sample size calculations

Two publications (2, 8) discussed considerations for conducting sample size calculations and determining expected effect sizes. Swann et al. (8) stated that prediction of specific microbial

organisms to be modulated by a dietary intervention beforehand is often difficult if not virtually impossible (8). Even if such predictions were possible, interpretation of specific microbial group compositional changes in terms of host health effects remains challenging (8). Both publications noted a challenge is that thresholds defining biologically meaningful effect sizes for changes in gut microbiome features or that differentiate individuals vary depending on outcomes of interest, are generally not standardized and often are not known (2, 8). For those reasons, effect sizes and sample size calculations for diet-gut microbiome studies commonly rely on host biochemical or clinical traits (which usually serve as primary study outcomes) thought to be influenced by diet-gut microbiome interactions rather than gut microbiome-related variables (8). Nevertheless, Swann et al. (8) described several limited instances where sample size calculations may be based on a gut microbiome measure. For example, if measurement of bifidogenesis after ingestion of a test fiber is sufficient to establish selective fermentation, sample size calculations may be performed based on effect sizes and standard deviation for fecal *Bifidobacteria* measured via quantitative approaches (8).

1.5 Statistical Analysis Plan

No considerations or knowledge gaps were extracted.

1.6 Interindividual variability

No considerations or knowledge gaps were extracted.

1.7 Inclusion and exclusion criteria

Swann et al. (8) noted that while age is important to consider we still lack a good understanding regarding when the gut microbiome becomes fully adult-like, and that the gut microbiome of individuals >65yr is distinct from younger individuals in both composition and

function. Therefore, the impact of dietary interventions on microbial-host interactions and their downstream effects on health may differ across the lifespan (8).

1.8 Metadata to collect

No considerations or knowledge gaps were extracted.

2. Diet intervention and assessment

A summary of the results for the diet intervention and assessment category is provided in **Table 2** and **Figure 3** of the main text and **Supplementary Table 3**. Below we summarize the extracted considerations and knowledge gaps.

2.1 Intervention design and development

Three publications (2, 4, 6) discussed factors to consider when designing/developing dietary interventions. Within those publications, multiple challenges inherent in designing interventions for diet-gut microbiome studies, particularly those using **whole food** interventions, were identified. Klurfeld et al. (4) stated that, relatively unique to this field, both nutritive and non-nutritive compounds (e.g., polyphenols, emulsifiers, etc.) modulate the gut microbiome and therefore influence health outcomes. Additionally, factors like food structure, preparation method, delivery mode and matrix can influence diet-gut microbiome interactions, but to what extent is often unknown (4). As a result, dietary ingredients and food preparation methods require careful consideration when designing interventions for diet-gut microbiome studies. Hughes et al. (2) emphasized that those considerations extend to development of the placebo/control condition and make it particularly challenging to develop a strong control for studies with whole food(s) as a dietary intervention. Further, designing placebos/controls that allow for isolation of diet-microbiome interactions as a causal factor driving changes in host phenotype (versus microbiome-

independent effect of the intervention) can be particularly challenging and requires careful consideration (4).

Inherent in designing any intervention is selecting the dose to be tested. Klurfeld et al. (4) encouraged researchers to consider side effects when selecting dosing stages with certain microbiome-targeted interventions, such as **fermentable fibers**. An additional consideration is that minimum dose required to elicit effects on the gut microbiome may be undetermined and whether the provided dose should be based on absolute dose, energy intake or other factors (e.g., body weight) can be unclear (4). Mohr et al. (6) described several unique challenges that exist for studies using **probiotic** interventions. They noted that important factors to consider related to probiotic dosing include effect sizes produced in previous studies, type of formulation, persistence within the host, colonization within the gastrointestinal tract, manufacturing processes, and stability of the final probiotic product.

2.2 Describing diets and interventions

Three publications discussed factors to consider when describing dietary interventions (1, 3-4). When using whole diets as interventions, Johnson et al. (3) suggested providing information on food preparation and cooking methods, food matrices, ripeness of foods, presence of additives, emulsifiers and artificial sweeteners, organic/conventional farming methods, microbial composition of non-fermented foods, and microbial load of dietary patterns. When using fiber as intervention, Klurfeld et al. (4) encouraged researchers to consider reporting more than just the amount and the chemical type of fiber, which may be insufficient and hamper reproducibility given the complexity and diversity of similar fiber types. Choi et al. (1) discussed dietary pattern indices and their potential to be considered for describing diets in certain studies. The indices can capture multiple dimensions of diet, and often incorporate balance and variety (1). However, Choi et al.

(1) also described limitations to that approach, including that existing indices rely on expert knowledge that fluctuates over time, while data-driven dietary patterns can be difficult to generalize beyond the study population and may not correspond with expert consensus and general nutritional knowledge (1).

2.3 Diet standardization

Two publications included factors to consider when standardizing participant diets in diet-gut microbiome research (2-3). Several of those considerations pertained to the proposed best practice of "stabilizing habitual diet". One reason why diet stabilization has been proposed as an alternative to providing complete standardized diets is that providing diets can be expensive and burdensome for participants and research staff (2). Johnson et al. (3) also commented that switching participants onto an identical, standardized diet could impact the gut microbiome, which may obscure intervention effects. Also, the optimal composition of any standardized diet is undefined and the extent to which diet standardization would reduce interindividual variability in the gut microbiome is unclear (3). Therefore, when considering individual nutrients or dietary components, Johnson et al. noted that it may be advantageous to allow participants to continue eating their habitual diets (2).

2.4 Assessing adherence/compliance

Six publications discussed considerations for assessing compliance/adherence (3-8). Marques et al. (5) stated that as in any nutrition study, measuring adherence/compliance is important and assessment methods, to some extent, will vary based on the population, dietary intervention, and outcomes of interest. Shanahan et al. (7) highlighted that very few established biomarkers for diet-microbiome and microbiome-host interactions have been identified. Those that have been identified can be expensive to measure, and the validity, reproducibility, and sensitivity

of some is suboptimal (7). Nonetheless, several biomarkers were described as potentially being particularly relevant to diet-gut microbiome studies.

Klurfeld et al. (4) and Swann et al. (8) both agreed that for interventions using **dietary or functional fibers,** fecal or blood short-chain fatty acids (SCFA) concentration and fecal pH measurements can be potentially useful biomarkers (4, 8). Fecal SCFA concentrations, however, may not reflect in vivo exposure given that 95% or more are absorbed before elimination in the stool (4). Mohr et al. (6) described breath hydrogen as a potential dietary fiber fermentation marker, while Marques et al. (5) encouraged recovery of fecal fiber. Of note, Klurfeld et al. (4) explained that despite the availability of these methods, a gap is that there are no well-established dietary fiber biomarkers and researchers must frequently rely on dietary histories that are often inaccurate, lack the necessary detail, and are subject to recall bias.

Biomarkers for several other foods and nutrients were also discussed. Swann et al. (8) described urinary alkylresorcinol concentrations as a short/medium-term biomarker of **whole grains** and **cereal brans** (particularly rye and wheat) intake (8). Urinary and fecal ferulic acid or serum dihydroferulic acid were listed as possible markers of **rye bran** and **whole grain wheat** intake (8). Circulating trimethylamine N-oxide (TMAO) can reflect dietary **choline** intake and may be particularly relevant to diet-gut microbiome interactions influencing cardiovascular health (7). Increasingly, various **polyphenols** are being studied in the context of diet-gut microbiome interactions. Shanahan et al. (7) noted that for polyphenols with low bioavailability that cannot be measured in blood directly, principle microbial metabolites can be used to monitor compliance.

Several emerging methods were discussed as also potentially being useful for measuring adherence/compliance in diet-gut microbiome studies. These include meta-barcoding to evaluate

the plant component of diet (3, 7), metabolomics, metagenomics, and natural enrichment of stable isotopes (8).

2.5 Diet assessment methods

Four publications discussed considerations for diet assessment methods (1, 3, 7-8). Swann et al. (8) noted that in general, best practice recommendations for diet assessment should follow those for any nutrition study (as described in (7)) while being adapted to provide the level of detail needed to adequately describe diets and interventions within the context of diet-gut microbiome interactions (8).

Shanahan et al. (7) highlighted the need for tools and methods that facilitate less onerous, yet accurate dietary intake estimation, especially those that enable the measurement of nutrients in foods that are relevant to microbes and that estimate the nutrient availability at the luminal substrate-microbe interface. Similarly, Choi et al. (1) noted that connecting food servings to the quantity of nondigested components reaching the colon where it likely has the largest impact on the gut microbiome will require new approaches to dietary assessment and analysis. Also, towards those goals, Johnson et al. (3) stated that several technology-based methods that incorporate image capture, wearable devices and apps are in development but still require validation. Devices for sampling throughout the gastrointestinal tract have also been developed or are in development (3). Eventually these tools may enable researchers to follow a meal through the gut and collect data about that meal, as well as corresponding changes to the microbial community in situ (3). Unfortunately, while these approaches will have the potential to provide precise estimates of exposure to specific dietary components and interactions of those components with gut microbes, the technology is likely to be impractical for many studies, costly, and burdensome in the near-term (3).

2.6 Database selection

Three publications included factors to consider for database selection (1, 3, 7). Shanahan et al. (7) stated that one of the challenges of comparing collected dietary data to food composition tables is that although many dietary constituents relevant to diet-microbe interactions have been measured in foods, not all are readily quantifiable in human diets or have not been measured comprehensively across the food supply. For instance, traditional macronutrient analysis alone may fail to uncover diet-microbiome relationships (7) and many non-nutritive food items containing high amounts of small molecules such as polyphenols (e.g., spices and herbs) are underrepresented in dietary databases and software (1). Moreover, Choi et al. (1) noted that because food composition tables are based on chemical analysis of foods, they do not account for variations in nutrient bioavailability and absorption, which ideally would be accounted for in diet-gut microbiome studies. Another factor that is not considered in the food composition tables is particle size of the ingested food, which combined with other components of eating context and environment (e.g., meal preparation, amount of chewing, etc.) can influence the amount of a food or nutrient reaching the gut microbiome (1).

Finally, Johnson et al. (3) emphasized that there is significant variability in food databases used across studies. Even within the English-speaking regions of the Americas, United Kingdom, and Australia there is no single database that is being consistently utilized (3). Nutrient composition sources, analysis methods for specific nutrients, naming conventions used to identify foods, and food grouping structures vary between different databases, which makes comparison across studies and cohorts extremely difficult (3). Therefore, Johnson et al. (3) urged researchers to establish a shared food ontology that can harmonize dietary data collected in different global regions and by different tools.

2.7 Other considerations

Two publications discussed several considerations related to dietary data collection and analysis (3, 7). Johnson et al. (3) noted that a single stool sample may reflect different durations of diet history for different people due to differences in transit time and suggested that three days of recording intake should be sufficient to capture relevant short-term dietary factors affecting microbiome composition in a single stool sample. Similarly, Shanahan et al. (7) encouraged collecting multiple dietary records to capture variability in daily dietary intake, regardless of the additional participant burden. Finally, Johnson et al. (3) noted that timing of meals can also affect gut microbiome composition but is largely ignored in diet-gut microbiome studies.

3. Biological sample analysis

A summary of the results for biological sample analysis category is provided in **Table 3 and Figure 4** of the main text, and in **Supplementary Table 4**. Below we summarize the extracted considerations and knowledge gaps.

3.1 Biological sample collection

Five publications discussed factors to consider for biological sample collection (3-5, 7-8). Most of the best practices identified dealt with **fecal samples**, which are commonly used in diet-gut microbiome studies. Klurfeld et al. (4) cautioned that feces can only provide spatially fixed data and does not accurately reflect longitudinal or horizontal environments of the colon. Additionally, the extent to which fecal samples capture the diet-microbiome interactions most relevant to host health outcomes is still largely unknown. Marques et al. (5) noted that while fecal samples are generally considered as samples of the metacommunity, anatomically explicit samples, such as **mucosal biopsies**, are greatly enriched for a site-specific community and, for

some outcomes, may be more relevant for detecting host-microbiome interactions. Further, regardless of the sample location and type, samples are only snapshots that do not distinguish between permanent and transient strains (4).

The **fecal metabolome** is thought to reflect the metabolic interplay between diet, host, and gut microbiome. However, Swann et al. (8) noted that as fecal metabolites remain unabsorbed from the gastrointestinal tract, they may be unseen by the host metabolic system, therefore, **urine and blood samples** may be more reflective of diet-gut microbiome-host interactions. Swann et al. (8) also highlighted a caveat present with collecting serum/plasma samples, which is that most metabolites produced by the gut microbiome from the ingested foods appear in blood 5-7 hours after ingestion and are cleared rapidly thereafter, thus, overnight fasted blood samples may not be informative. It is also essential to ensure that the collected sample is in alignment with the outcome of interest. For instance, Swann et al. (8) suggested that for metabolomics analysis, 24-hr urine would more accurately reflect intake over the previous days compared to spot urine samples.

Several of the reviewed publications emphasized that if fecal samples are to be collected, the **timing** of sample collection, transport, storage, and processing are all important to consider as all can impact study results. Collecting the entire bowel movement may also be important as a covariate, to quantify total microbial load, and for some studies to ensure adequate sample volumes when multiple outcomes (e.g., metagenomics, metabolomics, biomarkers) will be measured. However, doing so often presents logistical challenges such as the need to collect and transport large sample volumes (3). Differences in collection and transport methods add variability to results (5) and if quick sampling and proper collection procedures are not in place, the data generated are not useful and possibly even misleading. Johnson et al. (3) suggested that if samples are not collected on site, fresh samples should be transported to the research facility quickly where study

personnel can process, aliquot, and store them. Alternatives to transporting fresh samples include having participants process and freeze samples at home or using preservatives that maintain sample integrity without cold chain storage (5, 7-8). Johnson et al. (3) noted that having participants process their own samples can be a challenge as the burden of collecting a whole bowel movement and aliquoting into cryovials or microcentrifuge tubes may be overwhelming, and an alternative is using tubes with a detachable scoop connected to the lid. However, even with that convenience, the requirement to process samples may remain a deterrent for potential participants and can limit the number of aliquots collected. Several publications suggested that using preservatives to store samples may preserve sample integrity when quick transport, processing and freezing is not possible (3, 5, 7-8). Several preservatives that can allow samples to be stored at ambient temperatures were described (3, 5) with 95% ethanol, RNAlater (Thermo Fisher) and the OMNIgene gut kit (DNA Genotek Inc) showing high reproducibility and stability. However, some preservatives may interfere with certain measurements and 95% ethanol may have advantages over other methods in that respect when multiple analyses (e.g., DNA, rRNA, metabolites) are planned (3). Whatever the method, providing clear instructions on hygienic sample collection and ensuring ease of sample handling is critical (8).

In terms of **frequency** of sample collection, one reviewed report suggested that a “large” number of samples is needed to reach valid conclusions (4), while another stated that multiple measurements may be necessary to provide an accurate understanding of the individuals’ microbiome (8). One publication suggested collecting multiple samples per time point or daily sampling throughout the study (3). Another suggestion was to collect samples at baseline, early, middle, and late points (7-8). Regardless of these recommendations, due to many staff- and budget-related constraints, most diet-gut microbiome studies collect a single stool sample per time point.

3.2 Fecal sample data to record

Two publications (3-4) discussed considerations relating to metadata to collect for fecal samples. Johnson et al. (3) noted that measuring and reporting transit time when possible is recommended given evidence that rapid or slowed transit times can affect gut microbiome composition (10-11). Aside from transit time, measuring fecal pH and moisture can be considered. Klurfeld et al. (4) described both as potential examples of cost-effective and relatively easy measurements that can serve as general outcomes and aid in data interpretation.

3.3 Microbiota analysis

3.3.1 Measurement methods

Seven publications (1-3, 5-8) discussed considerations relating to microbiota measurement and other related measurement techniques. Multiple viable options for gut microbiome compositional and/or functional analysis such as shotgun metagenomic sequencing, 16S rRNA gene amplicon sequencing, fluorescence in situ hybridization (FISH), qPCR and DNA microarrays among others were described (8). Shanahan et al. (7) urged making decisions on which method to use at the trial design phase, prior to participant recruitment and data collection and to keep the selected technique consistent across all aspects of sample processing and analysis. Consistency was described as absolutely crucial as different sequencing platforms and bioinformatics tools/parameters can considerably influence results (2, 8, 12-15). A brief overview of considerations discussed regarding several common gut microbiome assessment techniques is provided below.

One of the most used microbial sequencing techniques is **16S rRNA gene amplicon sequencing**. Advantages of the 16S methodology are that it is relatively low-cost and quick (3, 5) and provides a snapshot of overall microbial community composition (7). Disadvantages include

relatively low-resolution that does not accurately assign taxonomy beyond the genus level (3, 7-8), inability to differentiate between closely related organisms (3), restriction to bacteria and archaea only (7), potential to introduce bias based on the primers chosen (3), and generation of compositional (i.e., relative abundance) rather than quantitative data. As a result, 16S rRNA gene amplicon sequencing alone is not desirable when a detailed assessment or quantification of taxonomy or community function is needed (3).

FISH and **qPCR** techniques are both 16S-based and can also be used to target specific bacterial groups, as well as provide quantitative data (e.g., to measure absolute abundance) (8). FISH is the only method that allows for direct visualization of histological localization of microbes in tissue (8). Unfortunately, FISH and qPCR do not assess the entire community and like 16S gene amplicon sequencing, fail to capture taxa below detection thresholds (8).

Whole metagenomic shotgun sequencing and **shallow shotgun sequencing** are alternatives to 16S-based techniques. The main advantages of metagenomic sequencing is the approach provides species (and even strain) level resolution (3, 5, 8), can measure microbial organisms other than bacteria and archaea (e.g., eukaryotic species and viruses) (3), is less prone to amplification bias (3), and captures functional genes thus providing information on potential metabolic properties and functional shifts in addition to taxonomy (5-8). Disadvantages include high cost (2-3, 6-8), high analytical and computational burden, the need for special expertise to interpret the obtained data (2, 6-8), and the lack of quantitative data. Additionally, databases used for computational profiling may lack genomes for a large fraction of the intestinal microbiome and data interpretation is complex, since presence of a gene does not necessarily indicate the gene is being expressed (8). Another limiting factor is that there are no set recommendations for minimum sequencing depth for metagenomics analysis (5) and it is generally constrained by the microbial

"dark matter" (microbes not yet cultured in a laboratory setting) (7). Johnson et al. (3) suggested shallow shotgun sequencing as a more cost-effective approach to whole genome sequencing that can also provide accurate resolution at the species level.

DNA microarrays were described as the most comprehensive pre-designed method for gut microbiota assessment (8). The method utilizes oligonucleotide probes immobilized onto a glass slide to hybridize to complementary nucleotide sequences and provides phylogenetic identification and quantification (8). DNA microarrays are highly reproducible and can be useful for defining complex microbial signatures relevant for a dietary treatment or enabling sensitive detection of probiotic species used in an intervention (8). The disadvantages of this method include providing incomplete coverage of the ecosystem (8), high subjectivity to cross-hybridization (2), potential difficulty detecting low abundance taxa (2), and the limitation to identifying species that will hybridize to the probes provided on the slide (2).

Regardless of the methodology used, once the sequences have been identified, studies typically use the sequences to assign taxonomy. Shanahan et al. (7) cautioned that taxonomic assignment using operational taxonomic units (OTUs) can make comparisons across studies difficult because each analysis will result in a unique set of OTUs. A more recent approach is to identify exact amplicon sequence variants (ASVs). ASVs give better resolution than OTUs and increase reproducibility as the same ASVs can potentially be identified across studies (7). In addition, ASVs retain all unique sequences, removing only those determined to represent sequencing error, which improves resolution and reproducibility (7).

Methods related to microbiota functional activity were also discussed in several publications. Choi et al. described **metatranscriptomics** as a method that can provide information about gene expression, which can improve identification of important diet-gut microbiome-disease

relations (1). Shanahan et al. (7), however, pointed out a disadvantage of this method, which is that RNA has a short half-life, making timing of sample collection a major confounding factor. In addition, this method is technically difficult to execute and requires attention to specific sample collection and storage procedures (6). **Metabolomic phenotyping** is a method that can be useful for providing insight into the biochemical output of the gut microbiome and how these outputs impact host health (8). Suggested metabolites to consider in diet-gut microbiome studies included SCFA, secondary bile acids, products of amino acid breakdown (e.g., tryptophan metabolites) and polyamines among others (8). However, metabolite analysis, just like metagenomics methods, is constrained by the volume of the microbial “dark matter” (7).

3.3.2 Relative versus absolute abundance

Four publications (2, 4, 7-8) discussed considerations for relative and absolute abundance measures. 16S rRNA gene amplicon sequencing and shotgun sequencing only provide relative abundance data but assess the complete community while other methods quantify a portion of the community. Without quantification it is unclear whether changes in relative abundance are driven by a change in absolute abundance or by collinear relationships within the community (7). While methods are under development to improve quantitation, currently there are no efficient and low-cost methods for measuring absolute abundance within the entire community and the extent to which the total number of bacteria in the gut is important is somewhat unclear (4).

3.3.3 Diversity measures

Four publications (1, 5, 7-8) discussed factors to consider when measuring, analyzing and reporting microbiome or diet diversity measurements. Three publications agreed that high **microbiome α-diversity** (i.e., within-sample diversity) and gene richness are generally considered

desirable but do not equate to greater abundances of microbes performing beneficial functions and is not an absolute requirement for stability and resilience of the microbial ecosystem (5, 7-8).

3.3.4 Reporting taxonomy

Three publications (1, 7-8) discussed factors to consider when reporting taxonomy. Swann et al. (8) noted that reporting at the ASV/OTU or species level allows for granular reporting which is valuable given the metabolic variability of the microbiome but highlighted that inter-individual variability can complicate analyses as granularity increases. Reporting at higher taxonomic levels (e.g., genus, family, phyla) may overcome some of that variability allowing for identification of key trends but will also mask potentially important details because microbes within a taxonomic group can have different metabolic activities or differential responses to dietary substrates (8). It was also emphasized that taxonomy alone does not provide mechanistic insight into the effect of diet on microbial utilization of nutrients (7) given that metabolic functions are not always linked to taxonomic identity (7), the most highly differentially abundant taxa may not represent meaningful differences in function, and low-abundant taxa (which are routinely excluded from analysis) can influence function (1). Thus, reporting on taxonomy alone may not be sufficient for diet-gut microbiome studies and direct measurement of metabolites in combination with characterizing microbial profiles (taxonomy and genes or gene pathways) may be more informative for health outcomes (7). In addition, there is little consistency as to which classification or taxonomic levels are reported in diet-gut microbiome.

4. Analyzing, integrating and reporting diet and gut microbiome data

A summary of the results for the analyzing, integrating and reporting diet and gut microbiome data category is provided in **Table 4 and Figure 5** of the main text and **Supplementary Table 5**. Below we summarize the extracted considerations and knowledge gaps.

4.1 Microbiome data transformation and normalization

Considerations and gaps for data transformation and normalization were discussed in two publications (1-2). Choi et al. (1) emphasized that feature-level microbiome data, β-diversity metrics, and intakes of foods and food groups measured using dietary records and food frequency questionnaires (FFQs) are rarely normally distributed. As such, various data transformations may be needed to analyze diet and gut microbiome data depending on the statistical approach and study hypothesis. However, Choi et al. (1) also noted that transforming data is not always straightforward. For example, Hughes et al. (2) highlighted that while log-transformation of microbiome sequencing data is often recommended, this method requires a pseudo count for the many zeros in the data set, the choice of pseudo count can impact results and there is no consensus on what count should be used. Data normalization may also be required in some instances and a common technique is total sum scaling (TSS). The method divides the number of reads assigned to a specific taxon by the total number of reads in the sample providing a measure of relative abundance (2). However, while TSS is straightforward, it may introduce bias (2) and there is some disagreement as to whether normalization is required and, if so, the most appropriate normalization approach (16).

4.2 Statistical modelling

4..2.1 General characteristics and diet-microbiome data integration

Four publications (1-2, 4, 8) discussed considerations for statistical modelling. A comprehensive discussion of various statistical tests, metrics, and model fitting/performance tests

suitable for diet-gut microbiome studies can be found in Choi et al. (1) and Hughes et al. (2). Briefly, applying multivariate approaches to dietary data analysis in diet-gut microbiome research was discussed as a best practice by two publications. Choi et al. (1) explained that this is largely because multivariate approaches have the potential to improve integration of diet and microbiome data, strengthen diet–microbiome signatures, and help control for variation in microbiome composition introduced by nutrition studies with microbiome-focused outcomes. An advantage of these approaches is that direct comparison between microbiome communities and dietary patterns can be made, thus improving integration of diet and microbiome data (1). Additional advantages of multivariate ecologically based approaches can be to reduce the problem of overfitting models that results from including too many variables such as many dietary components or microbial taxa and reducing the issue of autocorrelation (2). In dietary data, autocorrelation can arise from foods commonly consumed together (e.g., salad and salad dressing) (1). Klurfeld et al. (4) discussed another example of autocorrelation using diets high in protein and fat, which are necessarily low in carbohydrate (often digestible and non-digestible carbohydrate) making it almost impossible to attribute causality to an individual macronutrient. Similarly, within microbiome datasets, autocorrelation can arise from bacterial guilds and genome interaction groups that increase and decrease in concert and the compositionality of the data (i.e., an increase in abundance of one taxon will proportionally decrease the abundance of another) (1). Finally, application of emerging methods such as machine learning in diet-gut microbiome research was encouraged in two publications (1, 8). The methods were described as potentially helpful to uncover various dietary patterns obtained from repeated evaluations from a large population, construct models, and make microbiome composition predictions by learning from training data (1, 8) and as complementing multivariate statistical approaches.

Several publications also noted that diversity metrics can be applied to dietary data (1, 7). The approaches discussed included diet quality and diet pattern analysis:

Diet quality [e.g., Healthy Diet Indicator (HDI), Healthy Diet Score (HDS), Healthy Eating Index (HEI), Alternate Healthy Eating Index (AHEI), Mediterranean Diet Score (MDS)] assessments can be used in cohort, case-control, cross-sectional, and longitudinal study designs and may help account for dietary complexity and interactions between dietary factors (7). An advantage of these indices is that many have been validated by relating index scores to a specific health outcome (7) and facilitate comparisons across studies using the same index (1), both of which may be advantageous for diet-gut microbiome studies. An additional advantage can be that the indices typically reduce multivariate dietary data to a single continuous variable, which can make statistical analyses more straightforward. The main disadvantage of diet quality indices is that the majority require additional dietary intake assessment (e.g., food records, 24-hr recall, FFQ, etc.) to calculate the final score (7).

Diet pattern analyses, also suitable for cohort, case-control, cross-sectional, and longitudinal study designs, are generally good at accounting for diet complexity and interactions between dietary factors and can be used as a covariate to determine whether the effects of a nutrient are independent of the overall dietary pattern (7). The disadvantages of these methods include that patterns are empirically derived from data and not from diet-health evidence and that the empirical process can include arbitrary decisions (e.g., food groups, number of factors/clusters to be retained) (7).

Choi et al. (1) noted that dietary pattern analysis facilitates identification of diet-microbiome relationships in the context of dietary patterns and food groups, which is relevant because single nutrients are rarely consumed in isolation and food groups associate more strongly

with the gut microbiome compared to conventional nutrients. Food-based dietary patterns analysis avoids some limitations inherent in nutrient databases in which representation of food-derived biomolecules is lacking, and incorporates the concept of food synergy, that is foods may behave differently when consumed in combination rather than individually (1). Relatedly, Choi et al. (1) also discussed applying metrics originally meant for ecological assessment to measure diet diversity (as described in the statistical modeling section above). For example, hierarchical structures used in different food and nutrient databases can provide tree-like, lattice or hierarchical structures that organize food data and inform analysis of dietary patterns using approaches analogous to UniFrac, which is commonly used for microbiome analysis (1). The approach has potential to allow for analysis of multiple levels of dietary patterns, foods, and nutrients along with food synergy somewhat simultaneously (1). Development of a metabolite-informed tree structure for foods that accurately represent the diversity of food components in a hierarchical way without dependence on expert-defined food grouping structures or hierarchies could further improve this approach (1). However, appropriate ways to implement ecological metrics in dietary data analysis and how to interpret the obtained results is still largely undefined (1) and work to define the optimal tree structure for this purpose is still needed (1).

4.4 Correction for multiple comparisons

Only one publication (7) discussed considerations for handling type I error. Shanahan et al. (7) emphasized that care must be taken not to overinterpret the significance of differences in relative abundance of individual ASVs/OTUs and that presence of "rare microbes" (e.g., present in some but absent in the majority) may lead to an increased risk of type I error.

4.5 Reporting and interpreting results

Three publications (1, 4, 8) discussed considerations for reporting and interpreting results. Choi et al. (1) discussed methods for reporting dietary data, which included providing gram weight of foods consumed or normalized food weights that account for typically consumed serving sizes and discussed methods for reporting ratios of nutrients to energy intake. Swann et al. (8) focused on considerations for reporting gut microbiome-related outcomes, stating that the typical endpoints reported are alpha/beta diversity, taxa richness, and relative abundance of individual taxa. Klurfeld et al. (4) noted that almost all journals have online versions and offer online Supplementary materials as an option, which allows detailed reporting of dietary data.

4.6 Data sharing

No specific considerations or knowledge gaps were extracted.

References

1) Choi Y, Hoops SL, Thoma CJ, Johnson AJ. **A guide to dietary pattern-microbiome data integration**. J Nutr. 2022 may 5; 152(5):1187-1199. doi: 10.1093/jn/nxac033.

2) Hughes RL, Marco ML, Hughes JP, Keim NL, Kable ME. **The role of the gut microbiome in predicting response to diet and the development of precision nutrition models – part I: Overview of current methods.** Adv Nutr. 2019 Nov 1; 10(6):953-978. doi: 10.1093/advances/nmz022.

3) Johnson AJ, Zheng JJ, Kang JW, Saboe A, Knights D, Zivkovic AM. **A guide to diet-microbiome study design.** Front Nutr. 2020 Jun 12; 7:79. doi: 10.3389/fnut.2020.00079.

4) Klurfeld DM, Davis CD, Karp RW, Allen-Vercoe E, Chang EB, Chassaing B et al. **Considerations for best practices in studies of fiber or other dietary components and the intestinal microbiome.** Am J Physiol Endocrinol Metab. 2018 Dec 1; 315:E1087–E1097. http://doi.org/10.1152/ajpendo.00058.2018.

5) Marques FZ, Jama HA, Tsyganov K, Gill PA. **Guidelines for transparency on gut microbiome studies in essential and experimental hypertension**. Hypertens. 2019 Nov 4; 74:1279-1293. https://doi.org/10.1161/HYPERTENSIONAHA.119.13079.

6) Mohr AE, Pugh J, O’Sullivan O, Black K, Townsend JR, Pyne DB et al. **Best practices for probiotic research in athletic and physically active populations: Guidance for future randomized controlled trials.** Fron Nutr. 2022 Mar 8; 9:809983. doi: 10.3389/fnut.2022.809983.

7) Shanahan ER, McMaster JJ, Staudacher HM. **Conducting research on diet-microbiome interactions: A review of current challenges, essential methodological principles, and recommendations for best practice in study design.** J Hum Nut Diet. 2021 Aug; 34(4):631-644. doi: 10.1111/jhn.12868.

8) Swann JR, Rajilic-Stojanovic M, Salonen A, Sakwinska O, Gill C, Meynier A, Fanca-Berthon P, Schelkle B, Segata N, Shortt C, Tuohy K, Hasselwander O. **Considerations for the design and conduct of human gut microbiota intervention studies relating to foods.** Eur J Nutr. 2020 Dec; 59(8):3347-3368. doi: 10.1007/s00394-020-0223.
9) Pallmann P, Bedding AW, Choodari-Oskooei B et al. Adaptive designs in clinical trials: why use them, and how to run and report them. BMC Med. 2018 Feb 28; 16 (29). https://doi.org/10.1186/s12916-018-1017-7.
10) Fukuyama J, Rumker L, Sankaran K, Jeganathan P, Dethlefsen L, Relman DA, et al. **Multidomain analyses of a longitudinal human microbiome intestinal cleanout perturbation experiment**. PLoS Comput Biol. 2017 Aug; 13:e1005706. doi: 10.1371/journal.pcbi.10057
11) Procházková N, Falony G, Dragsted LO, Licht TR, Raes J, Roager HM. **Advancing human gut microbiota research by considering gut transit time**. Gut. 2023 Jan;72(1):180-191. doi: 10.1136/gutjnl-2022-328166.
12) Claesson, M., Clooney, A. & O'Toole, P. **A clinician's guide to microbiome analysis.** Nat Rev Gastroenterol Hepatol. 2017 Oct; 14(10):585–595. https://doi.org/10.1038/nrgastro.2017.97.
13) Knight R, Vrbanac A, Taylor BC, Aksenov A, Callewaert C, Debelius J et al. **Best practices for analysing microbiomes.** Nat Rev Microbiol. 2018 Jul; 16(7):410-22. doi: 10.1038/s41579-018-0029-9.
14) Sinha R, Abu-Ali G, Vogtmann E, Fodor AA, Ren B, Amir A et al. **Assessment of variation in microbial community amplicon sequencing by the microbiome quality control (MBQC) project consortium.** Nat Biotechnol. 2017 Nov; 35:1077. doi: 10.1038/nbt.3981.
15) Washburne AD, Morton JT, Sanders J, Mcdonald D, Zhu Q, Oliverio AM et al. **Methods for phylogenetic analysis of microbiome data.** Nat Microbiol. 2018 Jun; 3:652– 661. https ://doi.org/10.1038/s4156 4-018-0156-0.
16) McMurdie PJ, Holmes S. **Waste not, want not: why rarefying microbiome data is inadmissible.** PLoS Comput Biol. 2014 Apr; 10(4):e1003531.doi:10.1371/journal.pcbi.1003531.

Supplementary Tables

Supplementary Table 1. JBI Critical Appraisal Checklist for Text and Opinion Papers.

yes/no/unclear/not applicable (N/A)	Klurfeld	Johnson	Swann	Choi	Shanahan	Marques	Hughes	Mohr
Is the source of the opinion clearly identified?	Yes	Yes	Yes	Yes	Yes	Yes	Yes	Yes
Does the source of opinion have standing in the field of expertise?	Yes	Yes	Yes	Yes	Yes	Yes	Yes	Yes
Are the interests of the relevant population the central focus of the opinion?	N/A	N/A	N/A	N/A	N/A	Yes	Yes	Yes
Is the stated position the result of an analytical process, and is there logic in the opinion expressed?	Yes	Yes	Yes	Yes	Yes	Yes	Yes	Yes
Is there reference to the extant literature?	Yes	Yes	Yes	Yes	Yes	Yes	Yes	Yes
Is any incongruence with the literature/sources logically defended?	Yes	Yes	Yes	Yes	Yes	Yes	Yes	Yes

Supplementary Table 2. Best practice recommendations for study design and participant selection reported in diet-gut microbiome literature.

Category		Best practice	Reference
**Research questions and aims**		Should be hypothesis driven and all aspects designed to answer a question regarding a specific diet-microbiome interaction.	7
		Should consider mechanisms, interventions, and outcomes.	6
		Clearly state questions/aims at the outset.	8
**Trial design**		Should be guided by the hypothesis.	7
		Trial design must consider dietary intervention, resources available, and responses being measured.	2
		Utilize randomized controlled trial design if possible.	2, 4, 6, 8
		Favor longitudinal studies with a crossover design over parallel designs.	3, 5
		Parallel group design may be more suitable for probiotic studies.	6
		Combining human and animal models may be more informative than either approach alone.	4
		Include both microbiome- and host-specific assessments if aim is to demonstrate an intervention can confer health benefits to the host via interactions with the microbiome.	8
**Blinding**		Blinding should be used when possible.<br>Double-blinding (participants and researchers) is considered the gold standard.<br>Triple blinding (participants, researchers and data analysts) may be best for probiotic clinical trials.	3, 8, 6<br><br>6
		Report if blinding was used and if successful.	5
**Duration**	*run-in*	≥3-5 days*[1]	3
	intervention	Will depend on the primary outcome measure and, for probiotic studies, the specific strain utilized. 4-16 weeks common for probiotic studies.	6
		2-3 weeks so that gut microbiome can stabilize to intervention diet.	5
	washout	Determine based on follow-up assessments and ensure duration is sufficient to prevent carryover effects.	3, 6
		≥2 weeks	4, 6
		≥4 weeks	5
**Effect size**		Should be based on clinically relevant effects or standardized health recommendations where possible. Biological significance should be considered.	2, 5
**Sample size calculations**		Utilize largest sample size possible**	7
		400-500 for case-control and cross-sectional studies.	3
		Primary outcomes should be determined by the central research question. Metric should be dependent on the type of intervention and its intended effect, should be hypothesis-driven and may be based on biochemical, chemical, and physiologic traits rather than microbiome.	8
		Should consider intrinsic human gut microbiome variation.	5
**Statistical analysis plan (SAP)**		Statistical hypothesis and general features of primary variable analysis must be in place before submission for ethical approval. Detailed plan should be finalized before recruitment and sample collection.	7-8

<table>
<tr><td colspan="2" rowspan="1"></td><td>- Review and update after blind review of data and communicate to all relevant parties before blinding break.
- If microbial-related variable is the primary/secondary endpoint, analysis methods cannot be changed after unblinding.</td><td>8</td></tr>
<tr><td colspan="2" rowspan="2">Interindividual variability</td><td>Minimize impact by increasing sample size if appropriate and feasible. Otherwise, collect repeated samples to address temporal variability and reduce within-person noise.</td><td>3, 7</td></tr>
<tr><td>Utilize participant baseline microbiome as control if feasible.</td><td>5</td></tr>
<tr><td colspan="4"></td></tr>
<tr><td rowspan="16">Inclusion and exclusion criteria (factors to consider)***</td><td rowspan="3">general characteristics</td><td>Consider and/or match groups based on baseline dietary patterns and ability to maintain habitual diet if possible.</td><td>6, 8</td></tr>
<tr><td>Sequence gut baseline microbiome to enroll predicted responders if feasible.</td><td>3, 8</td></tr>
<tr><td>Case-control designs: maintain temporal, geographical, and demographic consistency in participant recruitment.</td><td>5</td></tr>
<tr><td rowspan="2">demographics/ anthropometrics</td><td>BMI, ethnicity</td><td>5</td></tr>
<tr><td>Age</td><td>5, 8</td></tr>
<tr><td rowspan="3">consumption of -biotics</td><td>Probiotic use</td><td>5-6, 8</td></tr>
<tr><td>Prebiotic use</td><td>6, 8</td></tr>
<tr><td>Fermented foods (in some cases)</td><td>6</td></tr>
<tr><td rowspan="2">washout for -biotics consumption</td><td>3–4 weeks</td><td>6</td></tr>
<tr><td>4-6 weeks</td><td>5</td></tr>
<tr><td rowspan="2">medication use</td><td>Antibiotics</td><td>5-6</td></tr>
<tr><td>Antifungal/antiviral, other drugs that may affect gut microbiome (e.g. Metformin)</td><td>5</td></tr>
<tr><td rowspan="2">washout for medication use</td><td>3-4 weeks</td><td>6</td></tr>
<tr><td>3-6 months</td><td>5</td></tr>
<tr><td rowspan="2">other</td><td>Comorbidities/pre-existing conditions</td><td>5-6</td></tr>
<tr><td>Weight loss programs, special diets (e.g., vegan, paleo, gluten-free, etc.), plan to change exercise level/intensity, recent long-distance travel, baseline microbiome composition</td><td>8</td></tr>
<tr><td colspan="4"></td></tr>
<tr><td rowspan="23">Metadata to collect</td><td rowspan="5">demographics/ anthropometrics</td><td>Sex/gender and age</td><td>3, 5-6, 8</td></tr>
<tr><td>Ethnicity</td><td>3, 5-7</td></tr>
<tr><td>Race</td><td>6</td></tr>
<tr><td>Cultural identification</td><td>5</td></tr>
<tr><td>Anthropometric data</td><td>5, 7</td></tr>
<tr><td rowspan="5">overall and GI health-related factors</td><td>Health status</td><td>7-8</td></tr>
<tr><td>Health history and diet-related GI symptoms</td><td>6</td></tr>
<tr><td>Recent illness, allergies</td><td>3</td></tr>
<tr><td>Primary disease and severity</td><td>7</td></tr>
<tr><td>Comorbidities</td><td>5, 7</td></tr>
<tr><td rowspan="5">supplement and medication use</td><td>Medication use</td><td>3, 6-8</td></tr>
<tr><td>Supplement use/history</td><td>3, 6</td></tr>
<tr><td>Probiotics</td><td>3, 7-8</td></tr>
<tr><td>Prebiotics</td><td>3, 8</td></tr>
<tr><td>Antibiotics</td><td>3, 7</td></tr>
<tr><td rowspan="4">diet</td><td>Habitual diet</td><td>3, 5-6, 8</td></tr>
<tr><td>Fiber intake</td><td>8</td></tr>
<tr><td>Coffee consumption</td><td>4</td></tr>
<tr><td>Alcohol consumption and water source</td><td>3</td></tr>
<tr><td rowspan="4">lifestyle-related factors</td><td>Lifestyle factors</td><td>6-7</td></tr>
<tr><td>Exercise/physical activity</td><td>3-4, 6, 8</td></tr>
<tr><td>Stress and smoking</td><td>4</td></tr>
<tr><td>Recent circadian disruptions</td><td>8</td></tr>
</table>

	geography/ environment	Geographic location/environment	5-6
		Urban/rural environment, birth location, immigration history	3
		Living structure, socioeconomic environment	5
	other	Recent or planned weight loss/gain	3
		Socio-environmental conditions (crowding, family composition and size), pets in the house	8
**Inclusion of diverse population**		When possible, be inclusive of all populations and utilize race/ethnicity as covariates in analysis.	6

[1]**Expert group comments:**
* Length of run-in will likely depend on study design and purpose of the run-in. A few days for run-in and washout periods may be appropriate for functional food/probiotic intervention studies where probiotic presence in the colon is the main outcome. For other studies this duration will likely be inadequate and for crossover studies specifically, washout should be at least the same length as intervention. Regardless of the selected duration, justification should be provided.
** Utilizing largest sample size may not be feasible due to associated cost. Sample size needed to answer the question of interest should be used. This can be determined via power analyses based on primary outcome variable.
*** Sex may be another important factor to consider in inclusion criteria.

Supplementary Table 3. Best practice recommendations for diet intervention and assessment reported in diet-gut microbiome literature.[1]

Category		Best practice	Reference
**Intervention design and development**	*diets/ foods*	Closely monitor foods/nutrients associated with outcome of interest and/or known to impact gut microbiome.	5
		Ensure participants maintain weight stability, unless study aim is related to weight loss.	5
		Control total amounts of dietary fiber, resistant starch, and fructans and other oligosaccharides.	4-5
		Consider dietary components that may act as microbiome modulators (e.g., macronutrient composition, certain micronutrients, non-nutritive compounds, polyphenols, probiotics, etc.).	8
	supplements	Probiotics:<br>- Ensure intervention is based on in vitro/preclinical and/or human studies and pharmacokinetics. Mechanism of action should align with primary outcome.<br>- Preserve in a live and robust state for entire study duration.<br>- Provide clear definition of intervention including strains, species, strain designations, dose frequency and duration given, formulation/mode of delivery.	6
		Probiotics:<br>- Before studying health benefits associated with probiotics in vivo, probiotic candidate strains should be characterized in vitro.<br>- Safety of probiotic must be demonstrated either by a history of safe use and/or specific in vitro and in vivo safety assessment prior to use in a human intervention study.<br>- Viable cell count and stability of count should be monitored and documented throughout the study.<br>All -biotics:<br>- Ensure compliance with specific characterization requirements of known microbial modulators when using probiotics, prebiotic fibers, and polyphenols as intervention.	8
	dose	For probiotic interventions:<br>- Determine based on previous work (e.g., clinical data) and gut survivability.<br>- Measure viability at start and end of study, add midpoint measurement for longer-term studies.	6
	placebo/ control	Incorporate placebo or control whenever possible.	2-3, 6, 8
		For interventions of isolated dietary fibers use microcrystalline cellulose.	8
		Whenever possible ensure placebo/control is indistinguishable from true intervention (e.g., physical appearance, taste profile, size) and has minimal effect on gut microbiome composition and/or activity.<br>- When not possible, present control as separate intervention and not true control.	2, 6, 8<br><br>2
		Ensure dietary intake/dietary pattern is equivalent among randomized groups.	5-6
**Assessing adherence/ compliance**	*biomarkers*	Measure relevant biomarkers when possible.	3, 5-8
		Relevant biomarkers:<br>- quantity of probiotic strains in fecal samples if intervention is probiotic<br>- urine alkyl resorcinol for whole grains<br>- urine/fecal ferulic acid or serum dihydroferulic acid for rye bran/whole grain wheat intake<br>- polyphenol metabolites (details reported elsewhere) (91).<br>- breath hydrogen	8

			6
		No established/reliable biomarkers currently available for fiber. May be relevant to measure fecal SCFAs or pH for fermentable fiber.	4, 8
		Blood samples should be timed to match pharmacokinetics of metabolite production.<br>- To capture potential effect of fermentable fiber on peak SCFAs concentrations blood samples can be timed for ~6 hours after consumption of intervention food.	5
	ensuring compliance	Should be assessed/monitored and methods reported.	5-6
		Collect uneaten intervention product and, if all meals are provided, uneaten foods/beverages to determine true intake.	3-5
		Have participants provide all items consumed to researchers so that food composition can be measured directly if consuming their own foods.	3
		Collect multiple food records (when diets are not fully provided).	3, 5
		Clearly explain study meal plans and ask participants whether they are willing to consume all foods as part of the screening process.	3
		Define acceptable levels of compliance a priori and exclude non-compliant subjects.	3, 8
**Describing diet/intervention**		Consider overall diet (e. g. global dietary parameters like diet quality and diet pattern) in addition to focusing on single nutrients or food categories if relevant for outcomes of interest.	1, 4, 7
		Provide as much detail as possible.<br>- For any intervention: trade names of the products used.<br>- For controlled feeding studies: brand names and amounts of all foods consumed.	4
		Divide foods into groups that have particular relevance to the gut microbiome based on fiber type or phytocompound composition known or suspected to affect the microbiome.	3
		Quantify dietary constituents relevant to diet-microbe interactions if possible (may be limited to those that are readily quantifiable in human diets and have been measured comprehensively across the entire food supply).	7
		Probiotics and synbiotics: identify phenotypic and physiological characteristics (from *in vitro* data). Report safety, purity, potency, viability, and stability. Name strains in accord with International Code of Nomenclature. Strains should be deposited in an internationally recognized culture collection.	6, 8
		Parabiotics: Report method of inactivation.<br>Postbiotics: Characterize metabolites and inanimate microbes.	6
		Isolated fibers/resistant starch and prebiotics: describe fiber source, CHO/sugar composition, purity, degree of polymerization, particle size, average molecular weight and its distribution range, glycosidic bonds, solubility, viscosity. Confirm resistance to digestion.	4, 8
		Isolated polyphenols: describe source, degree of polymerization, chemical structure, oligo-/polymer content and degree of polymerization.	8
**Diet standardization**		Select best dietary approach based on specific study characteristics (will vary). Ensure provided diets are consumed at the same time and location whenever possible.	3
		Provide diet the week prior to and throughout intervention, if possible. Otherwise, measure and report dietary intake.	6
		-Instruct participants to stabilize habitual diet rather than standardizing diet across participants. This can be done either by asking participants to maintain their normal diet (simple approach) or by assigning each subject to a specific constrained diet based on their own recent dietary intake data (more complex approach).<br>-Account for variability in dietary intake at baseline.	3, 8

		Standardized diets should be implemented if possible	2
**Methods**	*emerging methods*	Utilize technology and biochemical markers of intake as available. Emerging methods includes mobile and wearable technologies.	3
	commonly used methods	Select method based on nature of study, participant population, feasibility, strengths/limitations related to research question. Review specific best practice guidelines reported elsewhere (9).	6-8
		Combine multiple 24-hr recalls (AMPM method via ASA24) or 3–4-day food records (include 1 weekend day and use weighed records if possible) and FFQs.	3, 6
		Collect data in a way to allow for analysis of food choices and dietary patterns/quality as well as nutrient totals.	1, 3, 7
		Employ strategies to reduce under-reporting and/or other recall errors: provide clear instructions prior to completion of diet recording, utilize food models. Perform comprehensive cross-checking of collected data.	7
		- At a minimum, participants recording their intake should be trained using detailed examples showing the level of detail necessary to complete an accurate record (serving sizes, ingredient specificity, preparation methods, inclusion of commonly forgotten foods/additives).<br>- Include detailed longitudinal analysis of food intake that accounts for the multivariate nature of dietary data and relationships between foods.	3
		Provide food from metabolic kitchen and weigh all eaten/uneaten foods, else record everything eaten.	4
**Database selection**	*from nutrition- to microbiome-centered*	Report diet database utilized.	8
		- Avoid relying on existing nutrient composition variables as foods themselves are important when exploring diet-microbiome covariation.<br>- Connect dietary data to food databases that contain extensive information about foods and food components and use machine learning approaches to compare with microbiome data.	3
**Other considerations**	*timing with microbiome analysis*	Collect 2-3 days of diet recalls/food records prior to sample collection period.	3
		Time dietary assessment so that it is immediately adjacent to biological sample collection.	7
	timing & location of meal/supplement consumption	Record and report.	3, 5-6
		Keep meal timing consistent between participants if possible (e.g., at study center).	3

[1]**Abbreviations:** SCFA, short-chain fatty acids; CHO, carbohydrate; AMPM, automated multiple-pass; ASA24, automated self-administered 24-hr dietary assessment tool; FFQ, food frequency questionnaire.

Supplementary Table 4. Best practice recommendations for biological sample analysis reported in diet-gut microbiome literature.

Category		Best practice	Reference
**Bio sample collection*[1]**	*types of samples*	Should be appropriate to research question (e.g., stool vs. mucosa, small vs. large intestine).	7-8
		Mucosal biopsies are the gold standard if aiming to explore crosstalk between mucosal associated microbes and/or the intestinal epithelium and tissue-specific immune responses.	8
		Samples for microbiome analysis should be aligned with other sample types (e.g., blood).	5, 7
		Direct measurement of metabolites in combination with characterizing microbial profile may be more informative for health outcomes than taxonomic identification alone.	7
	quantity	Will vary based on outcomes of interest.	5
		A few grams often enough for deep shotgun sequencing and metagenomics	3
	frequency	Repeated microbiome sampling may help account for intra-individual variability. Collect 3-7 fecal samples per time point if possible.	3
		Collection and homogenization of 2-3 days' worth of fecal samples is recommended.	5
		Ideal to include baseline, early, middle, and late points during the intervention.	7-8
		Pre and post time points are a recommended minimum if study includes dietary intervention.	6
	timing	Record and report time and date of collection.	3-5, 8
		Use time of collection as covariate in analyses.	3
		Align collection with diet and biological assessment periods.	3, 7
		Record amount of time between sample collection and processing/freezing**.	5
	collection	Ensure procedures are standardized.	3, 7
		When samples are not collected in the laboratory/research center, home sampling with immediate freezing is recommended.	3, 8
		Participants can collect whole bowel movement using a container and a hat, transfer small amounts to storage tubes.	3
		An alternative to snap freezing is using solutions that preserve nucleic acid material (e.g. ethanol, RNAlater, OMNIgene Gut kit, DNA/RNA shield (Zymo).	3, 5
	transport, storage, and processing	Ensure procedures are standardized and appropriate for the type of sample and outcome of interest.	3, 5
		For microbiome analysis: freeze fecal sample immediately after defecation and store at -80C.	3, 5, 8
		Minimize freeze-thaw cycles to ≤ 1.	5, 8
		Maintain cold chain or use preservatives.	7-8
		For whole stool collection: ensure quick transport** to research facility.	3
		Mix fecal samples well if homogenization is required.	5
**Fecal sample data to record**		Transit time should be measured and recorded.	3-4, 8
		Record bowel movement frequency.	3, 5
		Record Bristol stool type.	5
	measurement methods	Should be based on research questions and align with resolution needed to address study hypotheses, resource availability, and features of microbiome (e.g., taxonomic, or functional resolution) being studied. Dietary intervention and response variables are especially important to consider.	2-3, 7-8

**Microbiota analysis**		- Non-template controls should be included during library prep to detect contaminant DNA.<br>- Use mechanical cell lysis prior to DNA isolation.	5
		If large numbers of samples need to be processed in batches or after a different length of storage, use controls to account for batch effects, and consider batch effects during statistical analysis.	7
		Use of mock communities to identify potential sources of bias.	8
		Ensure consistency in methodology across participants and throughout the study.	7-8
	relative versus absolute abundance	Measure and report both, especially if DNA yields vary between samples.	5, 7
		qPCR especially applicable to probiotic studies to measure fecal content of intervention strain(s).	6
	diversity measures	Specific metric should be reported.	5
		Consider assessing diversity metrics in combination with other measures of microbial community (e.g. structural stability over time, distinct phases of microbiome assembly, Principal Coordinates Analysis (PCoA), etc.).	
	reporting taxonomy	Report database and version used to assign taxonomy.	5, 7

[1]**Expert group comments:**
*If one wants to assess metabolites, transcripts, etc. the best practices for sample collection and storage may differ from those required for optimal microbiome sequencing
**Specific recommendations are provided elsewhere (11)

Supplementary Table 5. Best practice recommendations for analyzing, integrating and reporting diet and gut microbiome data reported in diet-gut microbiome literature.[1]

<table>
<tr><th colspan="2">Category</th><th>Best practice</th><th>Reference</th></tr>
<tr><td colspan="2" rowspan="4">Microbiome data transformation and normalization</td><td>Proportional and non-normally distributed data should be transformed (e.g., centered-log ratio).</td><td>7</td></tr>
<tr><td>Sequence count data often require normalization and/or rarefaction. Choice of method depends on data characteristics such as library size.</td><td>2</td></tr>
<tr><td>Rarefaction recommended for amplicon analysis (usually at least 10,000 reads/samples).</td><td>5</td></tr>
<tr><td>Apply rarefaction or use read count as an offset in statistical models if sequencing across samples is uneven.</td><td>8</td></tr>
<tr><td colspan="4"></td></tr>
<tr><td rowspan="10">Statistical modelling</td><td rowspan="7">general characteristics</td><td>Utilize models/tools designed specifically for microbiome analyses. Report model utilized.</td><td>5, 7</td></tr>
<tr><td>Use multivariate and mixed models to deal with confounding effects. Involvement of experienced statistician is important.</td><td>7</td></tr>
<tr><td>Statistical approaches that don't assume normality are essential and non-parametric approaches are needed. Non-parametric methods often better suited to microbiome taxonomy and beta diversity analyses.</td><td>1</td></tr>
<tr><td>Between group comparisons should be prioritized to evaluate effects of an intervention on the microbiome.</td><td>8</td></tr>
<tr><td>Prediction models:
- Testing a model in an independent cohort allows for broader assessment of the model (vs. internal cross validation) in the broad population.
- Which method to use depends on the nature and complexity of the data as well as the desired format of prediction.</td><td>2</td></tr>
<tr><td>Need to account for the error due to zero inflation.</td><td>1</td></tr>
<tr><td>When many zeros, statistical models based on negative binomial or Poisson distribution should be used for reliable identification of taxa that differ in abundance between groups.</td><td>8</td></tr>
<tr><td rowspan="3">diet-microbiome data integration</td><td>- Both dietary and gut microbiome data are compositional. Consider adjusting for compositionality or only analyzing relative changes.
- Adopting multivariate approaches for diet allows diet and dietary patterns to be treated as an additional "ome" for integration with other multi-omic data sets like microbiome sequencing data and fecal metabolomics.
- Once dietary patterns have been generated from food or food-group level data, then they can be passed to canonical correspondence analysis (CCA) and redundancy analysis (RA) to explore if the constraint of microbiome beta diversity distances by diet explains any amount of the variation in microbiome composition.
- Co-occurrence networks and co-abundance network analysis using both diet data at the food or food group level and microbiome features at the species or genera level with enough data density has the potential to reveal relations between dietary intake and specific members of the microbial community.</td><td>1</td></tr>
<tr><td>Variance partitioning, e.g., with permutational ANOVA (permANOVA) of beta-diversity provides a useful measure to quantify and rank order the sources of variation in the microbiome data to the intervention versus other effects.</td><td>8</td></tr>
<tr><td>3D modelling or "nutritional geometry" to simultaneously assess combinations of dietary components may prove useful in deciphering some of the complexity of interactions in diet-microbiome studies in humans.</td><td>7</td></tr>
<tr><td colspan="4"></td></tr>
<tr><td colspan="2" rowspan="2">Correction for multiple comparisons</td><td>Apply correction for multiple comparisons (e.g., Bonferroni, Benjamini-Hochberg, etc.) and report method used.</td><td>2, 5, 7</td></tr>
<tr><td>Utilize stringent filtering methods.</td><td>7</td></tr>
<tr><td colspan="4"></td></tr>
<tr><td colspan="2">Reporting of results</td><td>Limit conclusions to the ones derived from representative samples of controlled dimensions, and do not overinterpret results. Recognize biological relevance vs statistical significance. Variance explained in PCoA should be reported to determine biological relevance.</td><td>5</td></tr>
</table>

	Interpreting links between dietary intake and intestinal microbial community must consider substrate availability in the region sampled.	7
	Focusing on guilds or networks of co-abundant organisms that respond to the same dietary substrate may be informative.	7
	Positive and negative outcomes should be reported for transparency and confidence.	8
**Data sharing**	Sequencing data should be made available in repositories such as the National Center for Biotechnology Information (NCBI), Sequence Read Archive (SRA), and GitLab.	5
	Code relating to statistical tools used in the analysis should be made publicly available where possible.	5, 8
	Generate publicly available raw datasets. This will provide scope for meta-analysis of data regardless of original analysis techniques and metrics used for taxonomic levels reported.	7

[1]**Abbreviations:** ANOVA, analysis of variance

References

1) Choi Y, Hoops SL, Thoma CJ, Johnson AJ. A guide to dietary pattern-microbiome data integration. J Nutr 2022; 152(5):1187-1199.
2) Hughes RL, Marco ML, Hughes JP, Keim NL, Kable ME. The role of the gut microbiome in predicting response to diet and the development of precision nutrition models – part I: Overview of current methods. Adv Nutr 2019; 10(6):953-978.
3) Johnson AJ, Zheng JJ, Kang JW, Saboe A, Knights D, Zivkovic AM. A guide to diet-microbiome study design. Front Nutr 2020; 7:79. doi: 10.3389/fnut.2020.00079
4) Klurfeld DM, Davis CD, Karp RW, Allen-Vercoe E, Chang EB, Chassaing B, Fahey GC Jr, Hamaker BR, Holscher HD, Lampe JW, Marette A, Martens E, O'Keefe SJ, Rose DJ, Saarela M, Schneeman BO, Slavin JL, Sonnenburg JL, Swanson KS, Wu GD, Lynch CJ. Considerations for best practices in studies of fiber or other dietary components and the intestinal microbiome. Am J Physiol Endocrinol Metab 2018; 315:E1087–E1097. http://doi.org/10.1152/ajpendo.00058.2018
5) Marques FZ, Jama HA, Tsyganov K, Gill PA. Guidelines for transparency on gut microbiome studies in essential and experimental hypertension. Hypertens 2019; 74:1279-1293. https://doi.org/10.1161/HYPERTENSIONAHA.119.13079
6) Mohr AE, Pugh J, O'Sullivan O, Black K, Townsend JR, Pyne DB, Wardenaar FC, West NP, Whisner CM, McFarland LV. Best practices for probiotic research in athletic and physically active populations: Guidance for future randomized controlled trials. Fron Nutr 2022; 9:809983. doi: 10.3389/fnut.2022.809983
7) Shanahan ER, McMaster JJ, Staudacher HM. Conducting research on diet-microbiome interactions: A review of current challenges, essential methodological principles, and recommendations for best practice in study design. J Hum Nut Diet 2021; 34(4):631-644. doi: 10.1111/jhn.12868
8) Swann JR, Rajilic-Stojanovic M, Salonen A, Sakwinska O, Gill C, Meynier A, Fanca-Berthon P, Schelkle B, Segata N, Shortt C, Tuohy K, Hasselwander O. Considerations for the design and conduct of human gut microbiota intervention studies relating to foods. Eur J Nutr 2020; 59(8):3347-3368. doi: 10.1007/s00394-020-0223
